# Phosphatidylserine Synthase Controls Cell Elongation Especially in the Uppermost Internode in Rice by Regulation of Exocytosis

**DOI:** 10.1371/journal.pone.0153119

**Published:** 2016-04-07

**Authors:** Jin Ma, Zhijun Cheng, Jun Chen, Jinbo Shen, Baocai Zhang, Yulong Ren, Yu Ding, Yihua Zhou, Huan Zhang, Kunneng Zhou, Jiu-Lin Wang, Cailin Lei, Xin Zhang, Xiuping Guo, He Gao, Yiqun Bao, Jian-Min Wan

**Affiliations:** 1 National Key Facility for Crop Gene Resources and Genetic Improvement, Institute of Crop Science, Chinese Academy of Agricultural Sciences, Beijing, China; 2 School of Life Sciences, Centre for Cell and Developmental Biology, Chinese University of Hong Kong, New Territories, Hong Kong, China; 3 State Key Laboratory of Plant Genomics and National Center for Plant Gene Research, Institute of Genetics and Developmental Biology, Chinese Academy of Sciences, Beijing, China; 4 National Key Laboratory for Crop Genetics and Germplasm Enhancement, Jiangsu Plant Gene Engineering Research Center, Nanjing Agricultural University, Nanjing, China; Institute of Crop Sciences, CHINA

## Abstract

The uppermost internode is one of the fastest elongating organs in rice, and is expected to require an adequate supply of cell-wall materials and enzymes to the cell surface to enhance mechanical strength. Although it has been reported that the phenotype of *shortened uppermost internode 1* (*sui1*) is caused by mutations in *PHOSPHATIDYLSERINE SYNTHASE* (*OsPSS*), the underlying mechanism remains unclear. Here we show that the *OsPSS-1*, as a gene expressed predominantly in elongating cells, regulates post-Golgi vesicle secretion to intercellular spaces. Mutation of *OsPSS-1* leads to compromised delivery of CESA4 and secGFP towards the cell surface, resulting in weakened intercellular adhesion and disorganized cell arrangement in parenchyma. The phenotype of *sui1-4* is caused largely by the reduction in cellulose contents in the whole plant and detrimental delivery of pectins in the uppermost internode. We found that OsPSS-1 and its potential product PS (phosphatidylserine) localized to organelles associated with exocytosis. These results together suggest that *OsPSS-1* plays a potential role in mediating cell expansion by regulating secretion of cell wall components.

## Introduction

Cell division and anisotropic cell expansion determine the final size and shape of plant organs. Cell expansion involves “loosening” of existing cell wall architecture with synthesis and deposition of new cell wall components. Plant cell wall mainly compose of cellulose, hemicellulose, pectin, and structural proteins [1]. Cellulose is produced at plasma membrane (PM) by cellulose synthase complexes, while hemicellulose and pectin are synthesized and modified in Golgi and transported via vesicles to cell wall [2]. Mutations in genes associated with delivery of cellulose synthase genes result in altered cellulose contents and inhibition of cell elongation. CELLULOSE SYNTHASE INTERACTIVE1 (CSI1) is a microtubule-associated protein that bridges cellulose synthase (CESA) complexes and cortical microtubules, mutations in CSI1 cause defective cell elongation in hypocotyls and roots and reduce cellulose content [3]. Mutations in a PM-associated endo-(1→4)-β-D-glucanase disrupt normal wall assembly and cell elongation in both *Arabidopsis* [4] and rice [5]. Similarly, mutations in *COBRA* genes that encode glycosylphos-phatidyl inositol-anchored proteins are cellulose-deficient and compromised in organ elongation in Arabidopsis [6,7] and rice [8]. Xyloglucans and pectin are also involved in elongation growth. A recent study showed that improper secretion and accumulation of cell wall matrix polysaccharides including xyloglucan and pectin are associated with phenotypes of dwarf, curled rosette leaves, short petioles and short inflorescence stems [9].

In rice (*Oryza sativa* L.), elongation of internodes is controlled by cell division in the basal intercalary meristem and elongation of individual cells between nodes [10]. During the heading stage, rapid elongation occurs in the uppermost internodes (>5 cm per day; [11]). Several mutants defective in uppermost internode elongation have been identified in rice [12–15]. One of these mutants, *shortened uppermost internode 1* (*sui1*), is caused by mutations in *SUI1* that encodes phosphatidylserine (PS) synthase (PSS) [16,17]. In *Arabidopsis*, disruption of the gene encoding PSS reduces pollen fertility and plant height [18]. Mutations of *PSS* in *Schizosaccharomyces pombe* (*pps1Δ*) and the fungus *Candida albicans* (*cho1Δ/Δ*) lead to slow growth due to cell-wall defects [19,20]. These results imply an unidentified role of PSS in regulation of cell expansion.

The primary role of PSSs is PS biosynthesis. In bacteria and yeast, PSS catalyzes the conversion of CDP-diacylglycerol and L-serine into PS. In the endoplasmic reticulum of cultured Chinese hamster (*Cricetulus griseus*) ovary (CHO) cells phosphatidylserine synthase with base-exchange-type (BE-PSS) primarily utilizes phosphatidylcholine (PC, Cg-PSS1) or phosphatidylethanolamine (PE, Cg-PSS2) to synthesize PS [21]. As the most abundant negatively charged phospholipid in the cell, PS directs the binding of proteins with PS-recognition modules by electrostatic association of polycationic ligands with cellular membranes, as well as various physiological processes such as the coagulation cascade [22,23], recruitment and activation of signaling molecules [24], clearance of apoptotic cells [25,26], and vesicular trafficking [27–30]. The *cho1* mutant of *Saccharomyces cerevisiae* displays compromised PS synthesis and defected sporulation [31] and fusion of vacuolar vesicles [32]. In *Arabidopsis* and rice, PSS possesses enzymatic activity that catalyzes PC or PE into PS in yeast [17,18]. Verexpression of the wheat phosphatidylserine synthase gene induced necrotic lesions on *Arabidopsis* leaves [33]. However, the mechanism underlying the link between PSS and plant development remains to be clarified.

Here, we showed that mutation in *OsPSS-1* in rice leads to defected cell expansion and compromised cell wall biosynthesis. The shortened uppermost internode in *sui1-4* was mainly attributed to the disrupted secretion and deposition of cell wall components. Protoplast isolated from *sui1-4* mutant displayed a secretion defect. *OsPSS-1* was expressed predominantly in elongating tissues, and OsPSS-1 was co-localized dynamically with organelles associated with exocytosis. We also showed that mutation in *OsPSS-1* led to reduced PS contents. Our results revealed a potential role of OsPSS-1 in cell wall component trafficking.

## Results

### *sui1-4* is defective in cell expansion

**Fig 1:**
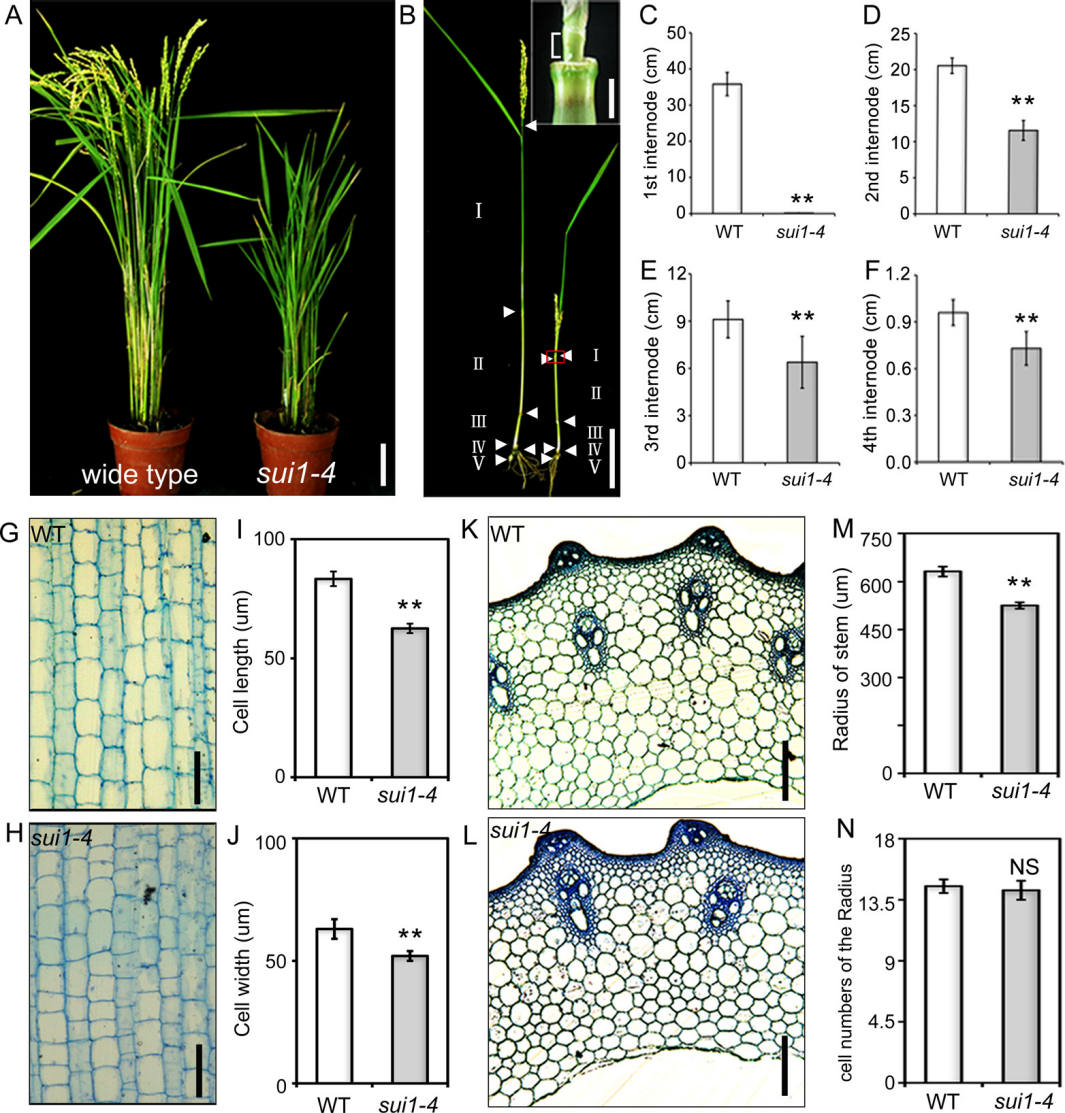
Phenotype of the *shortened uppermost internode 1–4 (sui1-4)* mutant. (A) Phenotype of the wild type (WT) plant and *sui1-4* mutant at the heading stage. Scale bar: 10 cm. (B) Internode lengths of the WT (left) and *sui1-4* (right) plants. Internode length was the distance between two adjacent nodes (white arrowheads). Inset: magnification of red rectangular section in (B), the uppermost internode (indicated by brackets) of *sui1-4* plants was shortened (Inset scale bar: 2 mm). Scale bar: 10 cm. (C) to (F) Internode lengths in WT and *sui1-4* plants. Differences were significant for each internode (**p<0.01, Student’s *t*-test). Error bars indicate standard deviation. (G) and (H) Longitudinal sections of the second internode of WT and *sui1-4* plants. Scale bars: 100 μm. (I) and (J) Cell length and cell width calculated from measurements of longitudinal sections of the second internode of WT and *sui1-4* plants. (K) and (L) Transverse sections of the second internode of WT and *sui1-4* plants. Scale bars: 100 μm. (M) Radii of stems calculated from measurements of transverse sections of the second internode of WT and *sui1-4* plants. Data are means ± standard error (n = 10). **p<0.01. (N) Comparison of cell numbers of radii in transverse sections of the second internode of WT and *sui1-4* plants (n = 10). NS, not significant.

A dwarf mutant was obtained from *japonica* cultivar (cv.) Kitaake. Seedlings of the mutant were slightly shorter than that the wild-type (WT) (S1 Fig). Dwarf phenotype in mature plants was attributed to reduced length of both panicles and internodes, especially the uppermost internode (Fig 1B–1F), accompanied with reduced fertility, decreased grain size and slightly increased tiller number (Fig 1A; S1C–S1H Fig). The mutant resembled the phenotype of other *sui1* mutants [16,17] and was designated as *sui1-4*. Investigations of the shortened second internode revealed slightly reduced cell length and width in longitudinal sections and cell radii of transverse sections, without any alternation in each cell number (Fig 1G–1L) indicating that internode cells in *sui1-4* plants had defective cell expansion.

**Fig 2:**
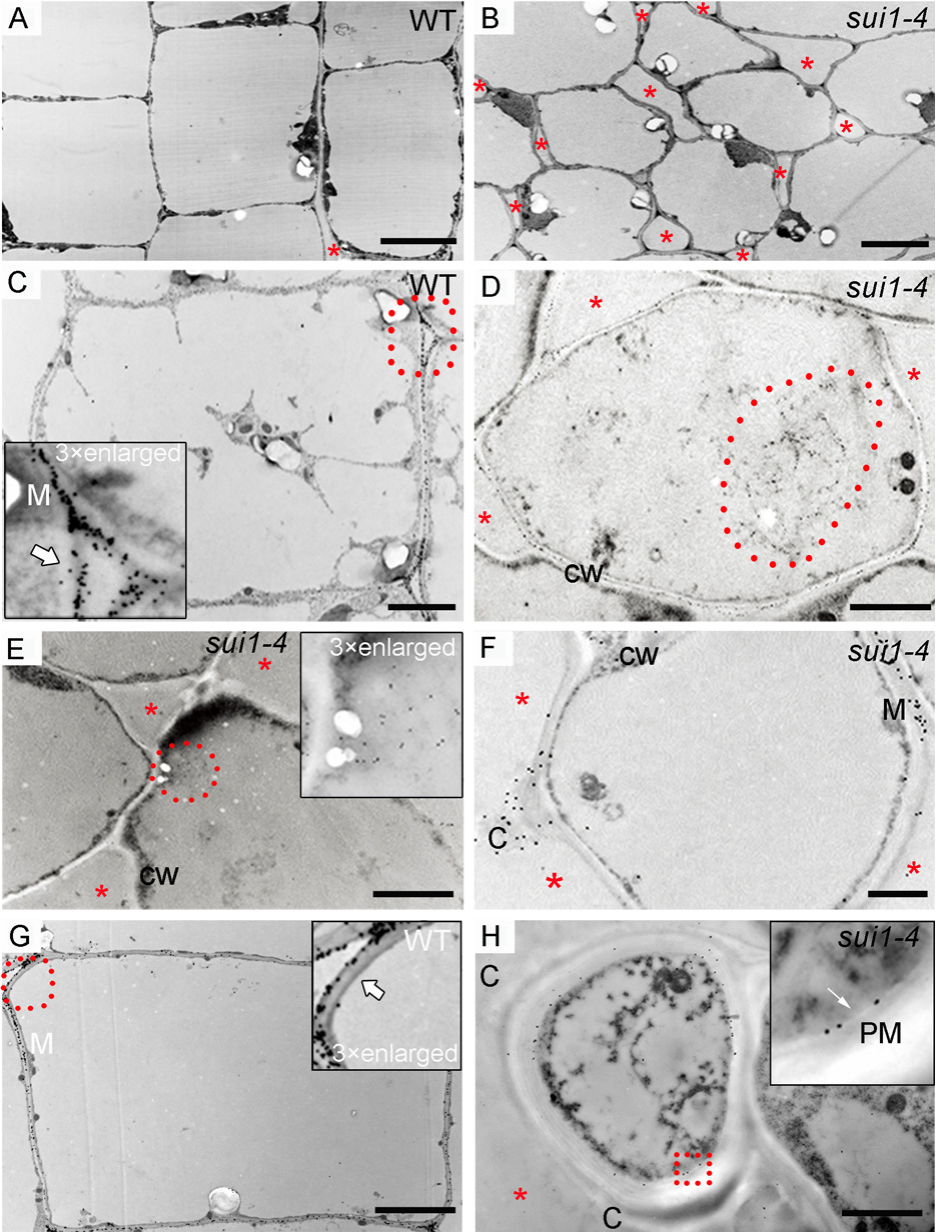
Pectin localization in the uppermost internode parenchymal cells of WT plants and *sui1-4* plants. (A) and (B) Transmission electron micrographs of parenchyma cell walls in the uppermost internode of WT (A) and *sui1-4* (B) plants. Note the non-elongated and loosely organized parenchymal cells (B) of the uppermost internode of the mutant compared to the well-developed cells in WT (A). Scale bars: 5 μm. In all panels, red asterisks denote intercellular spaces. (C) JIM7 signals scattered around the periphery of the intercellular space (white arrow) and concentrated toward the middle lamella (M) in WT plants. Inset: magnification of the area highlighted with a red dotted circle. Scale bar: 2 μm. (D) and (E) JIM7-tagged pectin clumps (red dotted circle) inside the cytoplasm. Inset in (E): magnification of selected pectin clumps. CW: cell wall. Scale bar: 2 μm. (F) JIM7 signal distributed in the middle lamella and in a clump (C) in a *sui1-4* plant. Scale bar: 2 μm. (G) JIM7-tagged pectin distributed in the middle lamella (M) and periphery of intercellular spaces (white arrow) in WT plants. Inset: magnification of the area highlighted with a red dotted circle. Scale bar: 2 μm. (H) As a negative control, clump could not been labeled by anti-PIP1s antibody which was predominantly present in the plasma membrane (white arrow). Inset in (H): magnification of selected red rectangle. PM: plasma membrane. Scale bar: 2 μm.

Dramatic phenotypic changes occurred in the uppermost internode of *sui1-4*. Cytohistological analyses showed that, compared to the organised longitudinal arrangement of cells in WT (S2A, S2C and S2D Fig), internode cells in *sui1-4* were loosely organised, with enlarged intercellular spaces and lacking in elongation in the uppermost intermodal region (S2B and S2E Fig). Transmission electron microscopy revealed that, compared to the longitudinally elongated and well-arranged parenchyma cells in WT plants (Fig 2A), parenchyma cells in the *sui1-4* were irregular in shape and had enlarged cell corners (Fig 2B).

### Cell-wall components are altered in *sui1-4*

Cell wall components were analyzed in *sui1-4* to find causes for the changes relative to WT. Cellulose contents in developed organs of *sui1-4* plants were lower than those in the WT plants (uppermost internodes, 12% lower; 2nd internode, 11.7%; leaf sheath, 8.3%). In 7-day-old seedlings, significant reductions in cellulose content were detected in root tips (4.6% less), and in leaves (9.2% less). The results suggested inadequate cellulose content in *sui1-4* plants (Table 1). We further examined other cell wall components in *sui1-4* plants, especially in the uppermost internode. The amount of glucose in cell-wall lysates, contributed from non-crystalline cellulose, xyloglucan and mixed linkage glucan, was reduced from 65.1 mg/g in WT plants to 35.7 mg/g in *sui1-4* plants. In contrast, both xylose and galacturonic acid (GalA), the major components of hemicelluloses and pectin originating in the Golgi body [34], were enriched in the uppermost internode of *sui1-4* (Table 1). Thus, cell wall components in *sui1-4* plants were quite different from WT.

**Table 1:** Monosaccharide composition of cell walls from various organs.

Tissue	Sample	Rhamnose	Fucose	Arabinose	Xylose	Manose	Galactose	Glucose	GalA	Cellulose
Uppermost internode	WT	2.1±0.0	0.8±0.5	28.6±0.5	281.2±5.4	1.6±0.0	7.4±0.1	65.1±1.8	8.1±0.0	545.2±5.4
	*sui1-4*	2.0±0.0	0.8±0.3	33.8±0.3*	389.1±5.7*	2.1±0.0	7.1±0.2	35.7±0.5*	23.0±0.5*	480.6±3.1*
2nd internode	WT	1.5±0.0	0.75±0.0	26.0±0.4	228.3±9.7	1.2±0.0	7.1±0.2	92.7±2.4	13.7±0.1	544.2±3.7
	*sui1-4*	1.7±0.0	0.76±0.0	26.2±0.7	220.8±4.6	1.2±0.0	8.0±0.2	82.0±1.3*	13.0±0.9	480.6±9.6*
Leaf sheath	WT	2.4±0.1	2.1±0.1	44.0±1.0	152.1±3.8	3.8±0.0	34.9±0.8	53.3±2.1	13.8±0.8	509.9±6.9
	*sui1-4*	2.7±0.1*	2.3±0.1	52.1±1.7*	166.7±4.0*	3.9±0.1	39.4±0.6*	55.7±2.0	13.8±0.3	467.4±7.6*
Root tips of 7-day seedling	WT	2.4±0.0	2.2±0.2	50.4±4.3	186.4±2.0	4.3±0.1	44.4±1.4	70.9±2.8	10.4±0.0	354.6±5.2
	*sui1-4*	2.4±0.0	2.3±0.0	52.6±2.5	218.7±4.0*	4.2±0.1	56.3±0.5	71.6±1.5	12.3±0.4	338.4±4.9*
7-day-old seedling leaf	WT	2.6±0.0	1.4±0.0	31.7±0.4	115.3 ±2.0	3.3 ±0.0	12.5 ±0.1	57.8±0.4	27.0±0.5	285.4±3.3
	*sui1-4*	2.7±0.1	1.3±0.0	34.2±0.8*	126.8±3.4*	3.2 ±0.0	12.3±0.2	55.0±0.7	28.7±0.6	259.2±4.2*

Alcohol-insoluble residues prepared from the uppermost internodes, 2nd internode, leave sheath, 7-day seedling of WT and *sui1-4* were subjected to compositional analysis. Results are calculated as μg/mg of alcohol-insoluble cell wall and are reported as the mean ± standard error of three independent assays. Significant differences between WT and *sui1-4* were determined with Student’s *t*-test (*p<0.01).

### Defective pectin secretion in the uppermost internodes of *sui1-4*

We surveyed the pectin distribution pattern of uppermost internode at the ultrastructural level by immunogold (15 nm gold) labeling using JIM7 antibody (15 nm in diameter) [35]. Pectins localize in primary cell walls, middle lamella, and cell corners, and play a major role in intercellular adhesion [36]. They are initially synthesized in a methyl-esterified form in the Golgi apparatus, and then delivered to the PM and cell walls by secretory vesicles [2]. Two weeks prior to heading, gold particles were restricted to the primary walls of WT parenchyma cells, especially to the middle lamella and periphery of intercellular spaces; there was no gold-labeled pectin in the cytoplasm (Fig 2C). However, in *sui1-4* cells, large amounts of gold-labeled pectins were observed in large compartments in the cytoplasm (Fig 2D and 2E; S3A and S3B Fig). One week prior to heading, although some gold particles were scattered in the middle lamella of *sui1-4* cell wall, most of the periphery of the intercellular spaces lacked gold labeling in comparison with well distributed gold particles in the intercellular spaces (Fig 2F and 2G). Additional, some pectin-rich “clumps” appeared in the outermost layer of the cell wall and protruded from the edge of enlarged intercellular spaces in the cell cap (Fig 2F and 2H; S3D Fig). To confirm these potential defects in pectin distribution, we performed high-pressure freeze substitution (cryofixation). Similar JIM7-labeled pectin clumps were observed in the outermost regions of the cell wall of *sui1-4* plants (S3E and S3F Fig), suggesting that the uppermost internodes were defective in pectin secretion and deposition.

### *sui1-4* plants exhibit compromised secretion

**Fig 3:**
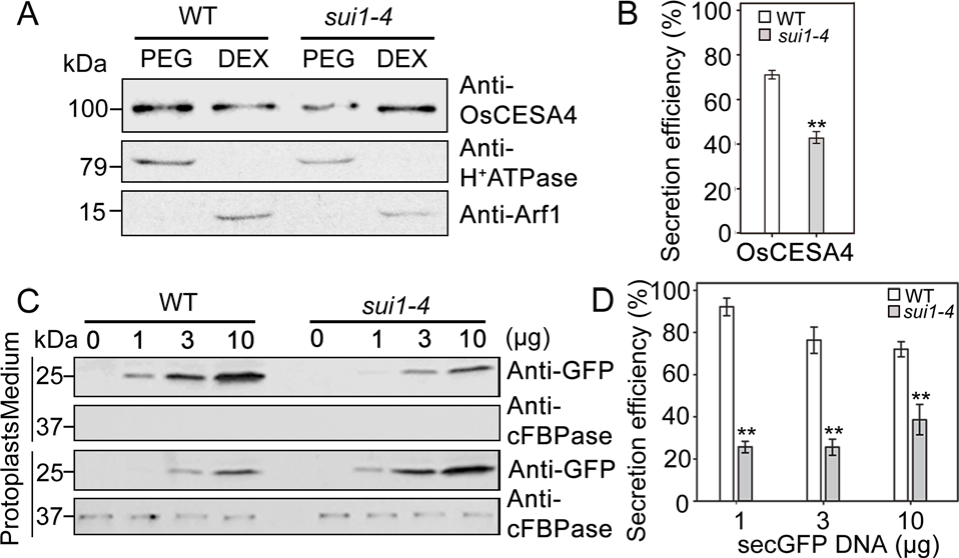
*sui1-4* is a secretion disorder mutant. (A) Fractions of membrane proteins extracted from 2-week-old WT and *sui1-4* plants immunoblotted with anti-OsCESA antibody. Anti-H^+^ATPase and anti-Arf1 were two marker proteins predominantly present in the plasma membrane and endomembrane fractions, respectively. The dextran (DEX) fraction contains endomembranes; the polyethylene glycol (PEG) fraction contains PMs. (C) Immunoblotting of secreted green fluorescent protein (secGFP) in WT and *sui1-4* protoplasts in a transient expression system. Equal amounts of protein extracted from protoplasts and culture medium were detected using anti-cFBPase, a marker for the plant cytosol. Immunoblotting was conducted in triplicate. 0, 1, 3, 10 (ug) represent the amounts of plasmid (SecGFP) when transient expression using rice protoplast. (B) and (D) Quantification of protein secretion in (A) and (C) relative to total protein (defined as 100%) using ImageJ. Three independent experiments were performed to obtain the average secretory efficiency. Differences were significant (**p<0.01, Student’s *t*-test). Error bars indicate standard deviation.

To determine if any soluble proteins or cell-wall-synthesis related enzymes have defective transport to the PM in *sui1-4*, we investigated trafficking of vesicles carrying OsCESA4, a member of the cellulose synthesis complex, which is believed to be synthesized in the endoplasmic reticulum (ER) and transported to PM via the trans Golgi network (TGN) [37,38]. We then investigated whether most of the synthesized CESA was transported from endo-membranes (indicated by dextran fraction) into PM (indicated by polyethylene glycol fraction) labeled by OsCESA4 polyclonal antibodies [39]. On fractional analysis to young seedling in WT and *sui1-4*, OsCESA4 mainly appeared in the polyethylene glycol fraction of WT plants, indicating its presence in PM, whereas in *sui1-4* plants, strong labeling was observed in the dextran fraction, reflecting its presence in endomembranes (Fig 3A and 3B). This result suggests that a large amount of OsCESA4 remained in the cytoplasm in the mutant, most likely due to failure in delivery to the PM.

To further evaluate the secretion defect, we employed a protoplast-based transient assay using a construct consisting of a secreted form of green fluorescent protein (secGFP) as an indicator [40]. We expected that secretion of secGFP to the apoplast may allow us to detect secGFP in the medium fraction, while perturbation of secretion would result in intracellular secGFP accumulation. Immunoblotting revealed that most of the secGFP was detected in the culture medium when protoplasts from the WT were used, whereas in protoplasts from *sui1-4* most secGFP was detected in protoplasts, with a very low amount detected in the medium (Fig 3C and 3D), suggesting an compromised secretion process in *sui1-4*.

### The *sui1-4* phenotype is caused by a mutation in *OsPSS-1* that is expressed predominantly in elongating tissues

**Fig 4:**
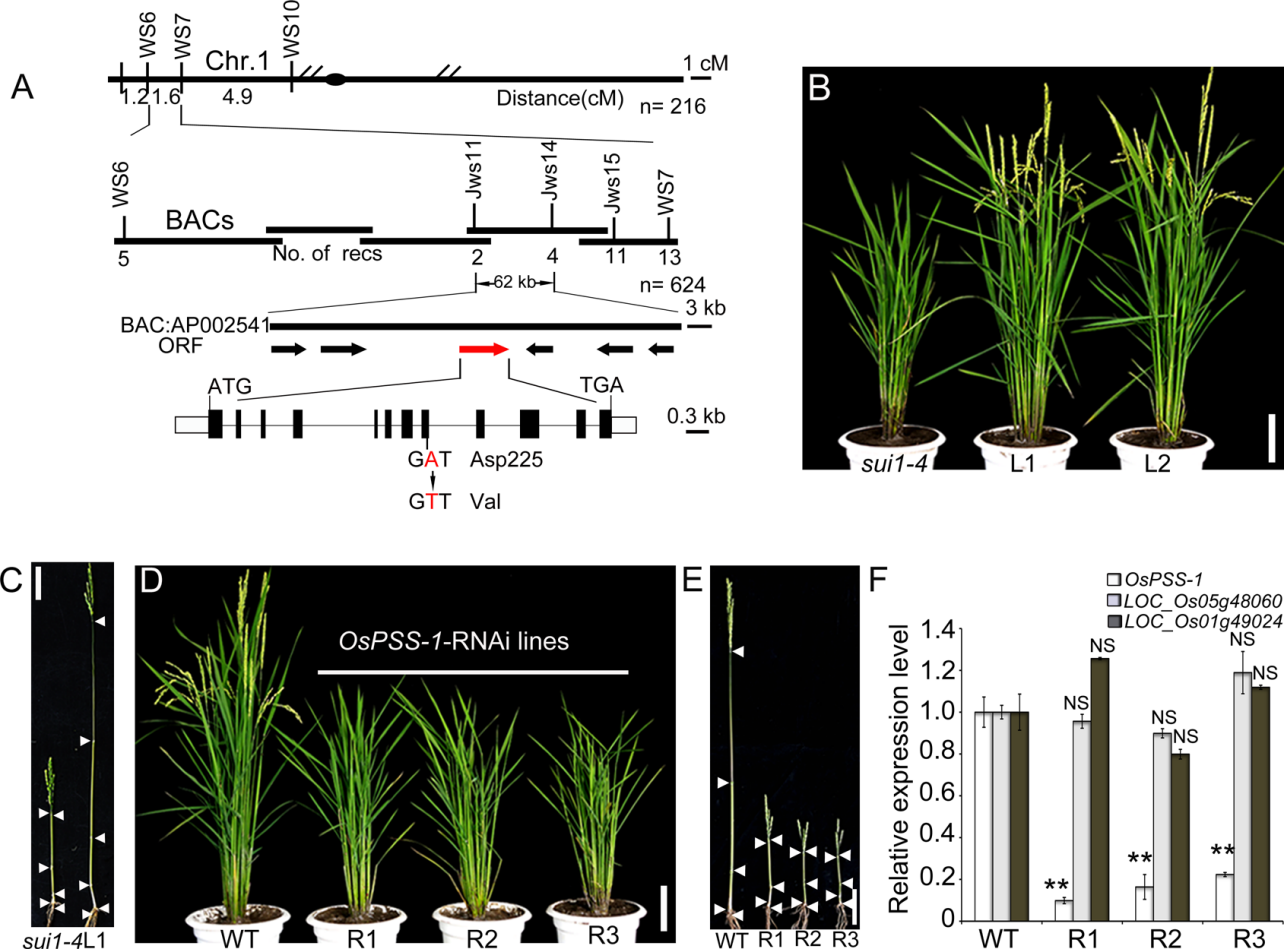
Positional cloning of *OsPSS-1*. (A) Fine mapping of the *OsPSS-1* locus. Molecular markers and number of recombinants (recs) are shown. BAC, bacterial artificial chromosome; ORF, open reading frame. (B) and (C) The WT genomic segment of *OsPSS-1* completely rescues plant stature. L1 and L2 denote plants from T1 transgenic lines. In (C), the nodes of the *sui1-4* mutant and L1 plants are indicated with white arrowheads. Scale bars: 10 cm. (D) *OsPSS-1-*RNAi transgenic lines mimic the phenotype of *sui1-4* plants. R1, R2, and R3 represent three independent T1 transgenic lines. Scale bar: 10 cm. (E) Internodes in WT, R1, R2, and R3 plants; white arrowheads indicate the nodes. Scale bar: 10 cm. (F) qRT-PCR reveals lower *OsPSS-1* expression in mutant plants than in WT plants, but transcript levels of OsPSS-1 homologs (LOC_Os05g48060 and LOC_Os01g49024) were not affected. Values are means ± standard error of three independent experiments. Significant differences were identified with Student’s *t*-test (**p<0.01); NS, not significant.

Genetic analysis revealed that the *sui1-4* phenotypes were inherited as a single nuclear recessive mutation (S2 Table). Map-based cloning of an F_2_ population made by crossing PA64 (an *indica* variety) with *sui1-4* revealed that the *sui1-4* phenotype was due to an A-to-T point mutation that converted a conserved Asp (position 225) to a Val in a putative PSS (designated as OsPSS-1; Fig 4A). Alignment of homologs from rice (LOC_Os01g02890, LOC_Os05g48060, and LOC_Os01g49024), *Arabidopsis* (AtPSS1), and maize (NP_001136592.1 and NP_001149567.1) revealed that PSS proteins are highly conserved (>80% similarity at the amino acid level; S4A Fig). OsPSS-1 contains eight transmembrane domains, as predicted by TMHMM Server 2.0, and the mutation occurred immediately after the start of the fourth transmembrane domain (S4B Fig). To verify that the mutation in *OsPSS-1* caused the phenotype, a construct containing 2,259 bp of the 5’ upstream sequence, the entire coding sequence, and 650 bp of the 3’ downstream sequence was transformed into *sui1-4* plants. Among the transgenic plants obtained, several lines with WT phenotype were identified, suggesting that the genomic DNA sequence used here was capable of complementing the *sui1-4* phenotype (Fig 4B and 4C). Thus, the mutation in *OsPSS-1* caused the *sui1-4* phenotype. When *OsPSS-1* expression was down-regulated while transcript levels of OsPSS-1 homologs were not affected using an RNA interference (RNAi) construct under the control of the ubiqutin promoter, transgenic plants displayed a phenotype that mimicked the *sui1-4* phenotype (Fig 4D–4F), suggesting that reduced *OsPSS-1* expression is sufficient to generate the dwarf phenotype.

**Fig 5:**
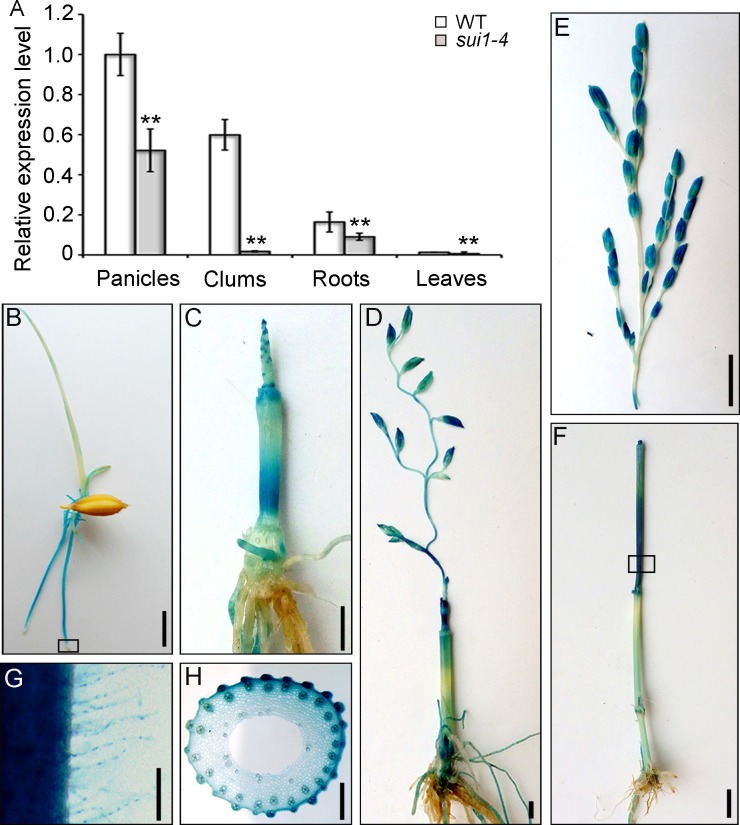
*OsPSS-1* expression and histochemical staining of *pOsPSS-1*::*GUS* transgenic plants. (A) qRT-PCR shows that *OsPSS-1* is ubiquitously expressed in various organs, with the highest level in panicles. For each organ, the *sui1-4* mutant displayed lower expression levels than WT plants. Values are means ± standard error of three independent experiments. Significant differences were identified with Student’s *t*-test (**p<0.01). (B) to (F) Histochemical staining of young seedlings one week after germination (B), at four weeks (C), one week before heading (D), and at the heading stage (E). Signals were detected in roots and coleoptiles (B), in young panicles and the basal regions of internodes (C) and (D), and divisional and elongating zones of the uppermost internode (E). The images in (E) and (F) are from two parts of the same transgenic plant. Scale bars: 1 cm in (B) to (D), 5 cm in (E) and (F). (G) Enlargement of histochemical staining boxed in (B). Scale bar: 100 μm. (H) Enlargement of hand-cut transverse section of the stem boxed in (E). Scale bar: 500 μm.

Quantitative reverse transcription polymerase chain reaction (qRT-PCR) analyses of WT plants revealed that *OsPSS-1* is expressed in various organs, including roots, culms, and leaves, with the highest expression in panicles (Fig 5A). We generated a reporter construct using a 2.7-kb fragment of the *OsPSS-1* 5’ sequence fused with the gene encoding β-glucuronidase (GUS). In young transgenic seedlings, GUS expression occurred predominately in the elongation zones of roots, root hairs, coleoptiles, and occasionally at the tips of newly formed leaves (Fig 5B and 5G). No expression was observed in shoot and root meristems. Four weeks prior to heading, strong GUS staining was found in young roots, the basal parts of internodes (divisional and elongating zones) originating from the intercalary meristem, and shoot apices (Fig 5C). Two weeks prior to heading, GUS staining shifted to the basal regions of the uppermost internode and the glumes (Fig 5D). During the heading stage, GUS expression was observed mainly in the uppermost internode (Fig 5E and 5F). Further examination of freehand sections of these tissues showed that GUS was ubiquitously expressed in all elongating cells, including parenchymal cells (Fig 5H). These results collectively suggested that *OsPSS-1* expression is necessary for cell elongation in various tissues.

### OsPSS-1 localizes to organelles associated with exocytosis

To elucidate subcellular localization of OsPSS-1, a construct was made with the OsPSS-1 C-terminal fused with the GFP N-terminal, expressed under the control of the CaMV 35S promoter (*p35S*: *OsPSS-1-GFP*), and transformed into the *sui1-4*. This construct rescued the *sui1-4* phenotype completely (S5A and S5B Fig). Fluorescence microscopy visualized strong GFP signals in the membrane network and punctate structures in root epidermal cells in *p35S*: *OsPSS-1-GFP* complemented *sui1-4* seedlings (S5C and S5D Fig).

**Fig 6:**
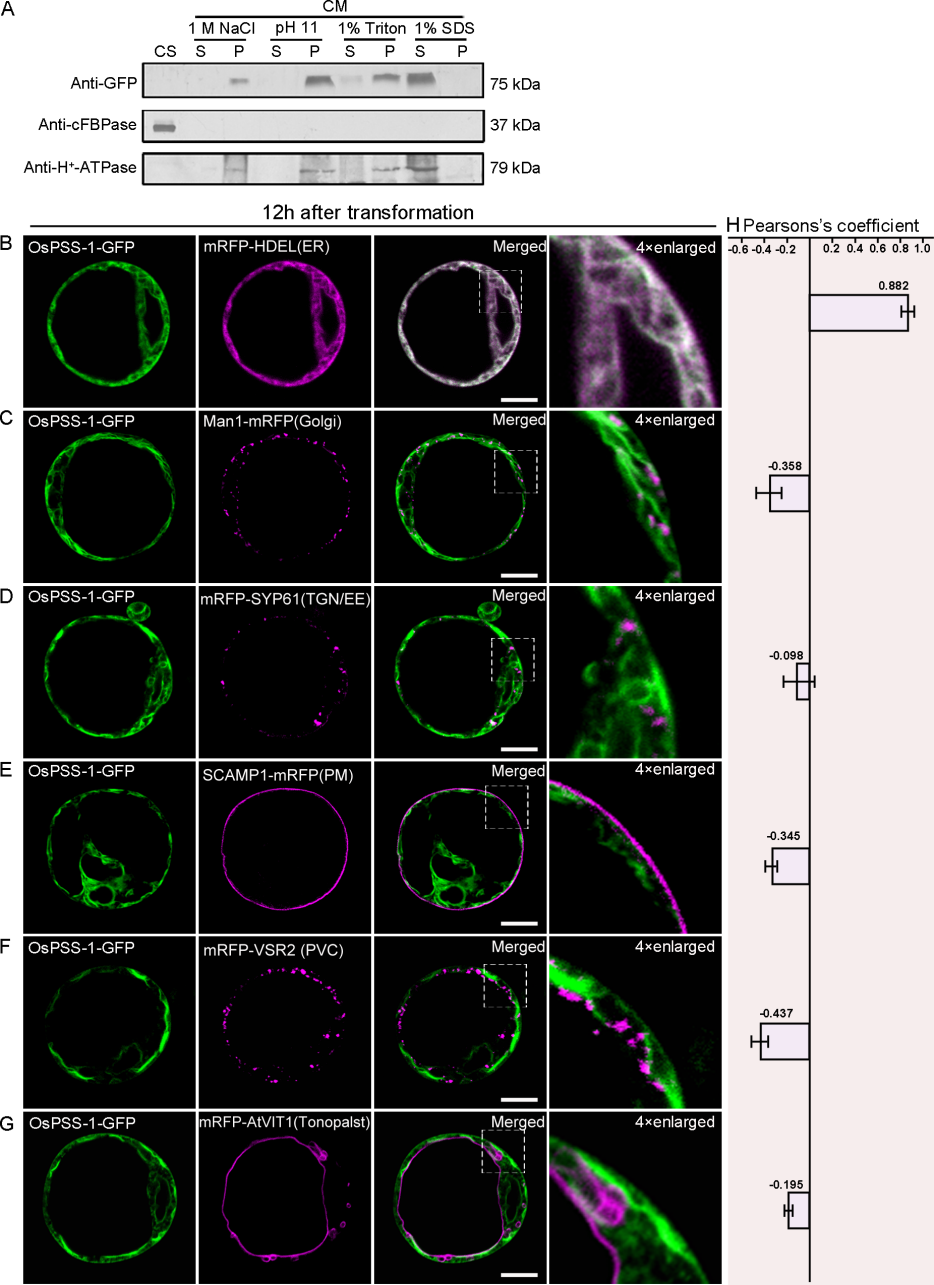
Subcellular localization of OsPSS-1-GFP 12 h after transformation in *Arabidopsis* protoplasts. (A) Immunoblot of proteins isolated from rice protoplasts expressing OsPSS-1-GFP. CM, cell membrane; CS, cell soluble; P, pellet; S, supernatant. (B) to (G) Confocal micrographs of the distributions of OsPSS-1-GFP (green) and indicated markers (magenta) 12 h after transformation. ER, endoplasmic reticulum; PM, plasma membrane; PVC, prevacuolar compartment; TGN/EE, trans-Golgi network/early endosomes. Scale bars: 10 μm. (H) For quantification, the PSC coefficients shown in the right panel (r_p_) were calculated after analysis of at least 10 individual protoplasts. The level of colocalization ranges from +1 for perfect correlation to -1 for negative correlation.

We purified the cytosolic and membrane protein fractions in transformed protoplasts isolated from WT seedlings. OsPSS-1 was primarily found in the membrane fraction, as indicated by staining with the membrane-specific antibody anti-H+-ATPase (Fig 6A). Most likely OsPSS-1 was an integral membrane protein because its presence in the supernatant depended on treatment with membrane-dissolving agents (1% sodium dodecyl sulfate (SDS) or detergent (1% Triton X-100)), but not with high salt (1 M NaCl) or high pH (pH 11) treatments (Fig 6A).

**Fig 7:**
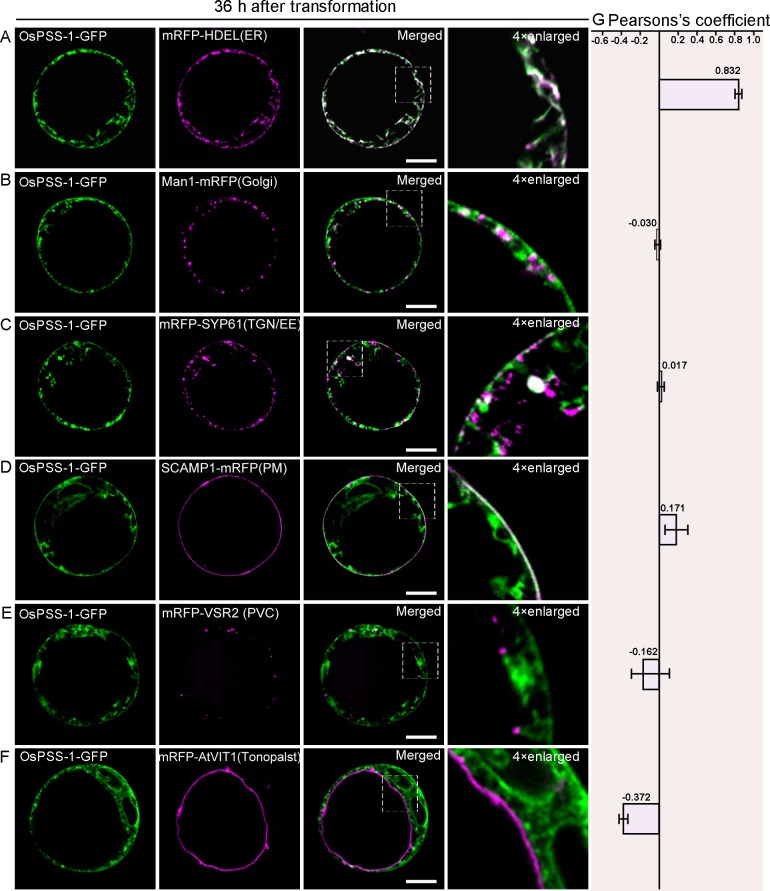
Subcellular localization of OsPSS-1-GFP 36 h after transformation in *Arabidopsis* protoplasts. (A) to (F) Confocal micrographs of the distributions of OsPSS-1-GFP (green) and indicated markers (magenta) 36 h after transformation. ER, endoplasmic reticulum; PM, plasma membrane; PVC, prevacuolar compartment; TGN/EE, trans-Golgi network/early endosomes. Scale bars: 10 μm. (G) For quantification, the PSC coefficients shown in the right panel (r_p_) were calculated after analysis of at least 10 individual protoplasts. The level of colocalization ranges from +1 for perfect correlation to 1 for negative correlation.

Further examinations in the WT protoplasts carrying *p35S*:*PSS1-GFP* suggests that OsPSS-1 is localized to the ER and PM (S5E and S5F Fig). To further elucidate the subcellular localization of OsPSS-1, a construct carrying the OsPSS-1-GFP fusion protein under control of the 35S promoter was co-transformed into *Arabidopsis* protoplasts transiently with constructs of monomeric red fluorescent protein (RFP) fused to either HDEL (an ER marker; [41]), ManI (a marker of the cis-Golgi apparatus; [42]), VSR2 (a marker of the prevacuolar compartment; [43]), SYP61 (a TGN/EE marker; [44]), VIT1 (a tonoplast marker; [45]), or SCAMP (a PM marker; [44]). When examined at 12 hours after transformation under confocal microscope, the OsPSS-1-GFP fluorescence was co-localized only with the ER marker HDEL (Fig 6B–6G). However, after 36 h of incubation, the ER co-localization was weakened, and additional green fluorescence appeared in some unidentified endomembrane compartments (Fig 7A). These compartments with green fluorescence were co-localized with the cis-Golgi apparatus (Fig 7B), TGN/EE (Fig 7C) and PM markers (Fig 7D). However, the OsPSS-1-GFP signal was independent from markers of the prevacuolar compartment (Fig 7E) and tonoplast (Fig 7F). Localization patterns were independent of the position of OsPSS-1 relative to GFP in the transformed construct (S6 Fig). Therefore, OsPSS-1 appeared to co-localize dynamically with the Golgi bodies, TGN/EEs and PM in accordance with the secretary pathway.

### PS has a similar localization pattern to OsPSS-1

**Fig 8:**
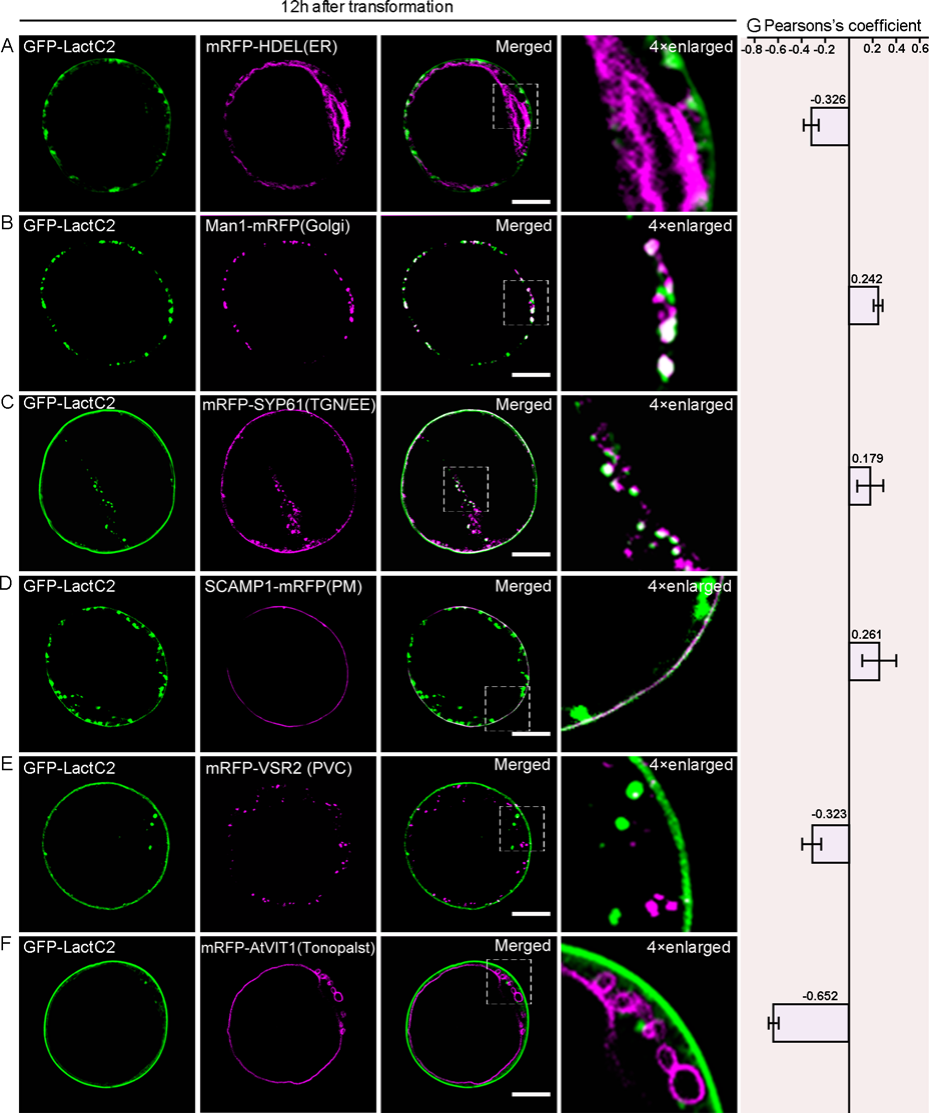
Subcellular localization of GFP-LactC2 12 h after transformation in *Arabidopsis* protoplasts. (A) to (F) Confocal micrographs of the distributions of GFP-LactC2 and indicated markers 12 h after transformation. ER, endoplasmic reticulum; PM, plasma membrane; PVC, prevacuolar compartment; TGN/EE, trans-Golgi network/early endosomes. Scale bars: 10 μm. (G) For quantification, the PSC coefficients shown in the right panel (r_p_) were calculated after analysis of at least 10 individual protoplasts. The level of colocalization ranges from +1 for perfect correlation to 1 for negative correlation.

Previously, PS could be detected with the biosensor Lact-C2 [28]. To inquire whether there is any difference in subcellular localizations of OsPSS-1 and PS, we observed the co-localization of PS with organelles that participate in secretion. Twelve hours after transformation, although no clear signal was visualized in the ER (Fig 8A) GFP-LacC2 signal was observed in the cis-Golgi (Fig 8B), the TGN/EEs (Fig 8C), the PM (Fig 8D), and absent from the PVC (Fig 8E) and the tonoplast (Fig 8F). The localization pattern of GFP-LacC2 was similar to that of OsPSS-1 36 h after transformation (Fig 7). The same subcellular localization patterns of PS were observed when GFP-LacC2 was transiently expressed in rice protoplasts from WT and *sui1-4* plants (S7 Fig).

### PS content is decreased in *sui1-4*

**Fig 9:**
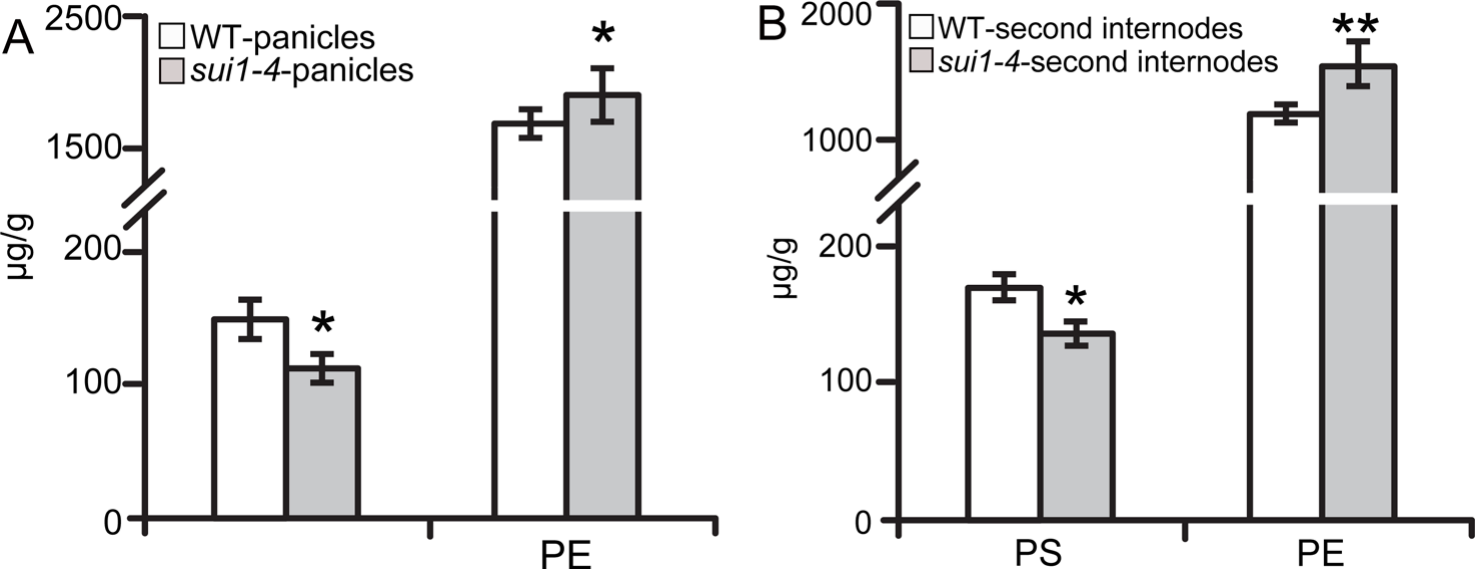
PS content is reduced in *sui1-4* plants. (A) and (B) Measurement of PS and its putative substrate PE. Values are means ± standard error of three independent experiments. (*0.01<p<0.05; **p<0.01).

Yin et al. inferred that OsPSS functions as PS synthase from co-localization of PS with the secretary pathway [17].We determined the PS contents in panicles and the second internodes in both the WT and *sui1-4* plants. The results showed that PS content in panicles was reduced from 151.2 μg/g in WT to 116.8 μg/g in *sui1-4* (Fig 9A), and in the second internode it was reduced from 171.3 μg/g to 142.9 μg/g (Fig 9B). Conversely, the content of the PSS substrate phosphatidylethanolamine (PE) was increased from 1,689.1 μg/g in WT panicles to 1,865.4 μg/g in *sui1-4* panicles (Fig 9A), and from 1,189.5 μg/g to 1,509.1 μg/g in WT and *sui1-4* in the second internodes (Fig 9B), suggesting that the mutation in *OsPSS-1* was associated with decreased PS content.

## Discussion

Cell expansion is a fundamental, dynamic cellular process driving plant growth through regulation of rate and direction. In this study, we showed that *OsPSS-1*, which is critical for cell expansion, is expressed predominantly in elongating cells. OsPSS-1 is similarly localized with PS in organelles involving exocytosis. Mutation in *OsPSS-1* leads the PS reduction, resulting in compromised secretion and defective deposition of cell wall components in *sui1-4* plants. Our results revealed a potential role of OsPSS-1 in mediating cell expansion and biosynthesis of cell wall components.

### Defective secretion of cell-wall components causes shortened uppermost internode

In stems, the architecture of the cell wall permits longitudinal elongation of cells while restricting radial expansion leading to highly asymmetric and anisotropic growth. The uppermost internode in rice is the fastest elongating organ during the heading stage [11]; consequently an adequate supply of cell-wall materials is essential in order to support such rapid elongation. Pectin, a highly heterogeneousmixture of polysaccharides, is a crucial component of cell-cell adhesion [46]. Mutations affecting pectin biosynthesis [36,47] and vesicular secretion [48] could cause intercellular attachment loss. In our study, cell wall components in the shortened uppermost internode of *sui1-4* were dramatically altered, including decreased cellulose levels and increased pectin and hemicellulose levels (Table 1). Reduced cellulose content was likely caused by compromised trafficking of CESA complexes to the PM as a large amount of CESA4 was accumulated in the endomembrane fraction of cytoplasm from the *sui1-4* plants (Fig 3A). Increased pectin and hemicelluloses contents would been explained by compensatory mechanism triggered by reduced cellulose content. The initial accumulation of pectin-containing compartments in the cytoplasm, and its eventual movement to the intercellular spaces (Fig 2), together with generally defective exocytosis indicated by secGFP secretion defect (Fig 3C), suggested that impairment of cell wall component secretion was the main factor causing cell elongation especially in uppermost internode.

### OsPSS-1 participates in exocytosis

Exocytosis occurs in most polarized, growing cells, including root hairs and pollen tubes of plants [49]. In rice, longitudinal cell elongation in roots and internodes adopts a similar, polarized growth pattern [50,51], indicating that exocytosis contributes to cell elongation. The results of GUS staining indicated that *OsPSS-1* is mainly expressed in rapidly elongating cells including roots and root hairs, especially in the uppermost internodes, rather than in meristems or fully developed tissues (Fig 5B–5F), corresponding to where exocytosis should be active, and suggesting that *OsPSS-1* expression is associated with exocytosis in elongating cells.

OsPSS-1 is an integral membrane protein. Its subcellular localization, which was established 12–36 h after transient transformation of protoplasts (Figs 6 and 7), suggested that OsPSS-1 is synthesized in the ER and transferred to the PM through the TGN, a pathway reminiscent of exocytosis. In cell wall-forming plant cells, exocytotic vesicles enclose cell-wall matrix precursors and host cellulose-synthase complexes in their membrane. Upon exocytosis, matrix materials are deposited in existing cell walls and CESA complexes are inserted into the PM [38].

Abnormal cellulose deposition indicated a possible trafficking defect in cellulose synthase complexes (CSCs). Small CESA compartments (SmaCCs) or microtubule-associated cellulose synthase compartments (MASCs) are critical for CSC recycling [52,53]. Recent work indicated that CELLULOSE SYNTHASE INTERACTIVE1 might be required for fast recycling of CSCs to the plasma membrane in Arabidopsis [54]. Detailed investigation on whether OsPSS-1 is specifically associated with SmaCCs/MASCs would be very informative in revealing the underlying mechanism.

### What is the role of PS in exocytosis?

In eukaryotic cells, membrane traffic and protein cargo delivery require accurate recognition and fusion of transport vesicles with acceptor membranes. As an important pathway of tethering post-Golgi secretory vesicles to the PM, exocytosis is mediated by the evolutionarily conserved exocyst complex, composed of Sec3, Sec5, Sec6, Sec8, Sec10, Sec15, Exo70 and Exo84 subunits [55]. Exocyst complex function relies on intricate interactions between exocyst and secretory vesicles, but importantly also between exocyst and specific membrane lipids [56]. In mammalian cells, PI(4,5)P2 (phosphatidylinositol 4,5-bisphosphate) has significant binding specificity with Exo70 over PS in mediating the targeting of exocyst to the PM [57]. PS is considered to be essential in mammals for the exocyst complex to combine with secretory vesicles [58]. In rice, three isoforms of PSS have been identified. *OsPSS-1* complements the phenotype of yeast PS-deficient mutant *cho1* [16,17] and the amount of PS is reduced in *sui1-4* (Fig 2), indicating OsPSS-1 potentially functions as a PSS to regulate phospholipid metabolism.

To investigate whether the exocyst complex was involved in the general secretion defect of *sui1-4*, we examined expression levels of exocyst subunit-encoding genes. Apart from the Exo70 subunit that consists of 47 paralogs in 9 clades (designated as *Exo70A* to *Exo70H*; [59,60]) other exocyst complex subunits in rice are encoded by either single genes or a few family members. The qRT-PCRs were performed for 12 selected culm-expressed *Exo70* paralogs and other exocyst subunit genes in *sui1-4* and WT. The results indicated that in young uppermost internodes of *sui1-4*, significantly reduced expression was detected with *OsSec5*, *OsSec6*, *OsSec8* and *OsSec10*. For the two-member families *OsSec3*, *OsSec15* and *OsSec84*, at least one gene had reduced expression level in *sui1-4* (S8 Fig). Of 12 *Exo70* paralogs tested, reduced expression was significant for *OsExo70A1*, *A3*, *B1*, *E1*, *F1*, *G1*, *H2* and *H3* in both young uppermost internodes (S8 Fig). Down-regulated expressions of exocyst subunits encoding genes forced us to determine whether OsExo70 subunits have potential of binding to the membrane lipids. Lipid-overlay assay showed that OsExo70 subunits could bound to PS in vitro (data not shown). Although there is no difference in PS localization due to OsPSS-1 between WT and *sui1-4* (S6 Fig), the less amount of PS existed in PM speculated by total reduced amount of PS perhaps affect targeting efficiency of the Exo70 subunits to PM in *sui1-4*. Whether PS content in PM was reduced in *sui1-4* compared to WT needs to be confirmed further. Take together, the results above implied that OsPSS-1 might play a critical role in exocytosis through PS. It will be interesting to explore whether other exocyst family members would interact with PS and the underlying mechanism of PS involvement in exocytosis.

## Materials and Methods

### Plant materials and growth conditions

*sui1-4* is a mutant derived from *japonica* cv. Kitaake. The mutant was crossed with *indica* cv. PA64 to construct an F_2_ mapping population. All rice materials used in this study were cultivated in the experimental fields of the Institute of Crop Science in Beijing or Sanya during the natural growing season or grown in pots in a greenhouse at 25–28°C with 16 h of light and 8 h of darkness.

### Map-based cloning of *OsPSS-1*

The *OsPSS-1* locus was mapped and cloned using 624 F_2_ individuals that exhibited the mutant phenotype. Primers for newly developed indel markers based on the comparison of genomic sequences from *indica* cv. PA64 and *japonica* cv. Kitaake (S1 Table) were employed to fine-map the mutated locus. For complementation analysis, the entire *OsPSS-1* coding region and its upstream and downstream sequences were amplified from WT genomic DNA using the primer pair OsPSS-1-HindIII-F/OsPSS-1-PstI-R (S1 Table). The resulting PCR fragment was ligated into the *Hind*III and *Pst*I sites of pCAMBIA1305 (Cambia) using an infusion cloning kit (Clontech) to generate pCAMBIA1305-OsPSS-1, which was introduced into *Agrobacterium tumefaciens* strain EHA105 via electroporation and into the rice mutant as described previously [61].

For RNAi, 539-bp fragments of *OsPSS-1* were amplified using primer pairs OsPSS-1-RNAi1-KpnI-F/R and OsPSS-1-RNAi2-BamHI-F/R (S1 Table). These fragments were separately cloned into *Kpn*I- or *BamH*I-digested sites in vector pCUbi1390-FAD2 (a ubiquitin promoter and a FAD2 intron inserted into pCAMBIA1390 [62]) using an infusion cloning kit to generate anti-sense and sense interference vectors.

### Sequence analysis and prediction of protein structure

Gene prediction was performed using the Rice Genome Automated Annotation System (http://ricegaas.dna.affrc.go.jp/). Sequences from rice, *Arabidopsis*, maize, human, and Chinese hamster were aligned using MacVector 12.0.2 (MacVector Inc.). Amino acid similarities among plant species were also calculated. The transmembrane domains of OsPSS-1 were predicted with TMHMM v2.0 (http://www.cbs.dtu.dk/services/tmhmm-2.0/).

### Gene expression analysis

Total RNA was extracted from various plant tissues using the RNAprep Pure Plant Kit (Tiangen). One μg of total RNA was reverse transcribed to cDNA using the SuperScript II Kit (TaKaRa) and primer pairs RTcF1/R1 (targeting *OsPSS-1*) and ActinF/ActinR (targeting the reference gene *Actin*). All qRT-PCRs were performed using the SYBR Premix Ex Taq Kit (TaKaRa) on a 7900HT Real-time PCR System (Applied Biosystems) according to the manufacturers’ instructions. The rice *Ubiquitin* gene was used as an internal control. Expression levels were calculated from three biological replicates, each consisting of three technical replicates. Gene-specific primers are listed in S1 Table.

For the GUS assay, 2.7 kb of *OsPSS-1* promoter sequence were amplified using the primer pair OsPSS-1-Pro-EcoRI-F/OsPSS-1-Pro-NcoI-R (S1 Table). The resulting construct, ProOsPSS-1∷GUS, was transformed into cv. Kitaake mediated by *A*. *tumefaciens*. Histochemical GUS staining was performed as previously described [63].

### Subcellular localization of OsPSS-1

To obtain GFP-expressing transgenic plants, *OsPSS-1* was amplified with primer pair 1305OsPSS-1-GFP-SmaI-F/R (S1 Table). The fragment was cloned into the *Sma*I site of pCAMBIA1305-GFP (produced by inserting the backbone behaving GFP from vector pAN580 into the *Sac*I-*Sal*I site of pCAMBIA1305) to yield vector pCAMBIA1305-D35S-*OsPSS-1*-GFP-NOS. This construct was introduced into WT and mutant plants to generate transgenic plants. Fresh roots from 2-day-old T_1_ seedlings were used for detecting GFP signals with confocal microscopy.

### Lipid measurements

Lipids were extracted from rice panicles and second internodes as described previously [18]. The phospholipids were dried by evaporation under nitrogen and stored at -20°C. Prior to analysis, samples were dissolved in chloroform. High-performance liquid chromatography was employed to measure phospholipid components. The analytical column was a YMC-Pack SIL-06 silica column (4.6 mm × 250 mm, 5 μm), and a Phenomenex silica column (3.0 mm × 4 mm) was used as a guard with the diode array detector at 205 nm. The temperatures for the column and detector were set at 30°C and 25°C, respectively. Twenty μL of the following solutions were injected into the column at a flow rate of 1 mL/min: solution A (chloroform:methanol:water:ammonium hydroxide 75:24:0.5:0.5) and solution B (chloroform:methanol:water:ammonium hydroxide 55:39:5.5:0.5). Solutions were used at 100% A~100% B at 0~20 min, 100% B at 20~40 min, 100% B~100% A at 40~41 min, and 100% A at 41–50 min.

### Chemical analysis of cell-wall components

Two grams of the uppermost internode from WT and *sui1-4* plants were extracted sequentially with ice-cold 80% ethanol, 100% ethanol, chloroform:methanol (1:1), and 100% acetone [64]. Destarched alcohol-insoluble residues were hydrolyzed in 2 M trifluoroacetic acid at 120°C for 90 min, then centrifuged. Supernatants were dried by evaporation under nitrogen and treated with sodium borohydride (10 mg/mL in 1 M ammonium hydroxide). After 24 h, the resulting alditol was extracted in ethyl acetate for analysis of monosaccharide composition with an Agilent 7890 gas chromatograph equipped with a 5975C mass selective detector (Agilent).

Pellets for cellulose analysis were treated with trifluoroacetic acid and hydrolyzed with Updegraff reagent. Cooled pellets were washed with acetone and hydrolyzed with 72% sulfuric acid. The anthrone assay was applied to measure cellulose content [65].

### Confocal laser scanning microscopy and transmission electron microscopy

For labeling of the cell wall, semi-thin (0.5 mm) sections were directly stained with calcofluor white (Sigma-Aldrich) for 5 min at room temperature, washed three times with phosphate-buffered saline, and subjected to confocal imaging with a Zeiss LSM700 laser scanning microscope. For transmission electron microscopy, intercalary meristem zones of *sui1-4* and WT plants were fixed overnight in 2% glutaraldehyde at 4°C, then exposed to 2% w/v OsO_4_ for 1 h. The samples were dehydrated under 30, 50, 70, 90, and 100% ethanol, infiltrated, and embedded in a low-viscosity medium with the Spurr Kit (Sigma). Samples were cut into ultra-thin sections (90 nm) using an Ultracut E Ultramicrotome (Leica) and mounted on formvar-coated copper grids. After post-staining with uranyl acetate and lead citrate, the sections were observed under a Hitachi H7500 transmission electron microscope.

High-pressure freezing and subsequent immunogold labeling were performed as described previously [66]. Immunolabeling of ultra-thin serial sections was performed using standard procedures [67] with JIM7 primary monoclonal antibody (purchased from the University of Georgia, USA) diluted 1:50 and anti-rat IgG-gold antibody (Sigma-Aldrich) diluted 1:20. For negative contrast, anti-PIP1s antibody was purchased from Agrisera.

### Transient expression analysis of *Arabidopsis* and rice protoplasts

WT *OsPSS-1* was amplified with primer pair OsPSS-1-GFP-BamHI-F/R (S1 Table). The fragment was cloned into the *BamH*I and *Bgl*II sites of vector PA7-GFP to produce constructs OsPSS-1-GFP and GFP-OsPSS-1, respectively. A LactC2 fragment was amplified from plasmid p416-GFP-LactC2 (Haematologic Technologies) using primers C2 domain-F/R (S1 Table) and inserted into the *Bgl*II site of PA7-GFP. OsPSS-1-GFP and GFP-LactC2 were co-transformed into rice or *Arabidopsis* protoplasts with various organelle markers and markers of the endomembrane system. Transient expression using *Arabidopsis* protoplasts from cell suspensions and rice protoplasts from rice leaf sheaths was described previously [68,69].

Confocal images were acquired at specific time points after transformation using an Olympus FV1000 system. For each experiment, more than 20 individual cells were imaged. For statistical analysis, the PSC colocalization plug-in [70] for ImageJ [71] was used to calculate the linear Pearson correlation coefficient (r_p_) of red and green fluorescent signals. We changed red fluorescent signal to magenta to make it easy for colorblind individuals to interpret the data in figures.

### Subcellular distribution of OsPSS-1 and CESA4

Whole protein extracted from WT protoplasts was separated into cell-soluble and cell-membrane fractions. Both fractions were subjected to protein gel blotting and were probed with anti-GFP antibody (1:2000 dilution, Sigma-Aldrich). Successful fractionation, which was determined by probing the fractions with anti-cFBPase (Agrisera) and anti-H+-ATPase (Agrisera), indicated that OsPSS-1 did not appear in cell-soluble fractions. To further investigate whether OsPSS-1 is an integral membrane protein, cell-membrane fractions were treated with high-salt (1 M NaCl) and alkaline (pH 11) buffers to extract peripheral membrane proteins, as well as detergent (1% [v/v] Triton X-100 and 1% SDS) to extract integral membrane proteins.

For CESA4, 2 g of fresh weight, 1-month-old plants were ground into fine flour using liquid nitrogen and homogenized in extraction buffer (25 mM Tris-HCl [pH 7.5], 2 mM ethylene diaminetetraacetic acid, 0.25 M sucrose, 2 mM dithiothreitol, 10% glycerol, 15 mM b-mercaptoethanol, and proteinase inhibitor cocktail). To separate the proteins into polyethylene glycol and dextran fractions, microsomal proteins obtained from supernatant ultracentrifuged at 100,000 × *g* for 1 h at 4°C were resuspended in fractionation buffer (5 mM K_2_HPO_4_–KH_2_PO_4_ [pH 6.8], 0.25 M sucrose, 1 mM dithiothreitol, 6.2% PEG3350, and 6.2% dextran T500) and centrifuged at 8,000 × *g* for 10 min at 4°C. Proteins in the polyethylene glycol and dextran fractions were separately collected and concentrated at 100,000 × *g* for 1 h and dissolved in suspension buffer (2 mM Tris [pH 6.5], 1 mM dithiothreitol, and 0.25 M sucrose). Approximately 9 μg of protein were subjected to gel blotting and detected with anti-OsCESA4 polyclonal antibody. Specificity of the anti-OsCESA4 antibody was previously confirmed [39]. For positive contrast, anti-Anti-H^+^ATPase and anti-Arf1 antibody was purchased from Agrisera. The immunoblots were quantified by measuring the intensity of the protein bands with ImageJ software.

### Secretion assay

Secretion of secGFP was assayed as described previously [72], with some modifications. After 14 h of transient expression, protoplasts and culture medium were collected separately via centrifugation at 200 x *g* at 25°C for 5 min. Proteins in protoplasts were extracted with 250 mM 2-amino-2-(hydroxymethyl)-1,3-propanediol (Tris)-HCl [pH 7.4], 750 mM NaCl, 5 mM ethylene diaminetetraacetic acid, and protease inhibitor cocktail, and sonicated for 5 s. The supernatant was recovered after centrifugation at 25000 × *g* at 4°C for 10 min. Proteins from the culture media were concentrated using a 3,000 MWCO Amicon Ultra 0.5 mL centrifugal filter (Millipore) at 1500 × *g*. Intracellular and medium proteins were then separated via SDS polyacrylamide gel electrophoresis and immunoblotting with anti-GFP (1:2000 dilution, Sigma-Aldrich) or anti-cFBPase (Agrisera, a control for protein loading) as indicated. Three independent experiments were performed to obtain the average targeting efficiency. The immunoblots were quantified by measuring the intensity of the protein bands with IMAGEJ software.

## Accession Numbers

Sequence data from this article can be found in the GenBank/EMBL databases under the following accession numbers. OsPSS: LOC_Os01g02890, LOC_ Os05g48060, and LOC_Os01g49024. AtPSS1: At1g15110. ZmPSS1 and ZmPSS2: NP_001136592.1 and NP_001149567.1, respectively. HsPSS1 and HsPSS2: UniProt accession numbers P48651 and Q9BVG9, respectively. CgPSS1 and CgPSS2: UniProt accession numbersQ00576 and O08888, respectively. Actin: Os03g0718100. Ubiquitin: Os03g0234200.

## Supporting Information

S1 Fig Phenotype of *sui1-4* and comparison of agronomic traits in WT and *sui1-4* plants. (A) Comparison of primary root growth kinetics in WT and *sui1-4* mutant seedlings. Each value represents a mean ± s.d. of 25 seedlings. (B) Comparison of four-weak-old seedings of WT and *sui1-4* mutant.(C) to (H) Differences in panicle length (C), spikelet fertility (D), 1000-grain weight (E), tiller number (F), grain length (G), and grain width (H). Data are means ± standard error. Significance was determined by Student’s *t*-test (*0.01<p<0.05; **p<0.01).
(TIF)

S2 Fig Confocal microscopy of longitudinal sections of the uppermost internode at the heading stage. Cell walls were stained with calcofluor white, a non-specific dye for β-glucanblue. The uppermost node and partially attached internode of WT plants (A) and entire uppermost internode of *sui1-4* plants (B) are shown. WT cells were well organized at the internode base (D) or elongated in the elongation zone (C), as indicated by white arrowheads in (A), compared to the disorganized, small cells with large intercellular spaces in the *sui1-4* mutant (E), as indicated by white arrowheads in (B). Red asterisk indicates an internode cavity. IB, internode base; N, node; IN, internode; P, panicle. Scale bars are 0.5 mm in (A) and (B) and 15 μm in (C) to (E).
(TIF)

S3 Fig JIM7 localization in the uppermost internode parenchymal cells of *sui1-4* plants. (A) and (B) JIM7-tagged pectin clumps (red dotted circle) inside the cytoplasm. Inset in (*B*): magnification of selected pectin clumps (red dotted circle). CW, cell wall. Scale bars: 2 μm. In all panels, red asterisks denote intercellular spaces. (C) JIM7-tagged pectin in the cytoplasmic flow or deposited ahead of the flow. Scale bar: 300 nm. (D) JIM7 signal distributed in the clump (C). Scale bar: 1 μm. (E) and (F) JIM7-tagged pectin clumps on immunoelectron microscopy of sections cut from a sample frozen under high pressure. Scale bars: 2 μm.
(TIF)

S4 Fig Structure and sequence analysis of OsPSS-1. (A) Multiple sequence alignments of the deduced amino-acid sequence of OsPSS-1 and its homologs. The beginning and ending sites of each domain are indicated above the sequences. Eight transmembrane domains were predicted in OsPSS-1 (red lines). Amino-acid residues critical for catalytic activity (open triangles), free serine binding/recognition (closed triangle), enzyme regulation (open circles), and enzyme production and/or stability (diamonds) are highly conserved. The amino acid (boxed by yellow line) mutation at position 225 (Asp→Val) in the *sui1-4* mutant is crucial for enzyme action or maintenance of the structure required for serine base-exchange activity. Accession numbers used for this alignment are given in Materials and Methods of the main text. (B) TMHMM v2.0 topology prediction for OsPSS-1. Eight transmembrane domains were predicted, with both the N terminus and a short C terminus facing the cytosol. Black arrow, position of the mutation in the *sui1-4* mutant.
(TIF)

S5 Fig Subcellular localization of OsPSS-1-GFP in transgenic rice root cells and protoplasts and *Arabidopsis* protoplasts. (A) and (B) The 35S promoter-driven *OsPSS-1*-*GFP* transgene rescues the phenotype of the *sui1-4* mutant. C1 denotes plants from T1 transgenic lines. In (B), white arrowheads indicate each node. Scale bars: 10 cm. (C) and (D) Confocal microscopy indicates that OsPSS-1-GFP is localized to the membrane network and to punctate structures in root epidermal cells of WT (C) and *sui1-4* (D) transgenic seedlings. Scale bars: 10 μm. (E) and (F) Confocal microscopy shows that OsPSS-1-GFP (green) colocalizes with the endoplasmic reticulum (ER) (E) and plasma membrane (PM) (F) markers in protoplasts from WT plants. Scale bar: 3 μm. (G) to (I) Subcellular localization of OsPSS-1-GFP (12 h (G) and 36 h (H) after transformation) and GFP-LactC2 12 h after transformation (I) in *Arabidopsis* protoplasts.
(TIF)

S6 Fig Subcellular localization of GFP-OsPSS-1 (fusion order reversed) in *Arabidopsis* protoplasts. (A) to (F) Confocal microscopy of the distributions of GFP-OsPSS-1 (green) and the indicated markers (magenta) 12 h after transformation. PM, plasma membrane; PVC, prevacuolar compartment; TGN/EE, trans-Golgi network/early endosome. Scale bars: 10 μm. (G) to (L) Confocal microscopy of the distributions of GFP-OsPSS-1 and the indicated markers 36 h after transformation. Scale bars: 10 μm.
(TIF)

S7 Fig Subcellular localization of GFP-LactC2 in WT and *sui1-4* protoplasts. Confocal microscopy reveals the same subcellular localization pattern in wild-type ((A) to (E)) and *sui1-4* ((F) to (J)) protoplasts (green, signal from GFP; magenta, signal from RFP). DIC, differential interference contrast; ER, endoplasmic reticulum; PM, plasma membrane; PVC, prevacuolar compartment; TGN/EE, trans-Golgi network/early endosome. Scale bars: 5 μm.
(TIF)

S8 Fig Expression analysis of exocyst complex subunits. (A) and (B) qRT-PCR of the genes encoding exocyst complex subunits in the uppermost internode of wide type and *sui1-4*. Significant differences were determined with Student’s *t-*test (*0.01<*P*<0.05; **P<0.01; NS, not significant).
(TIF)

S1 Table Primers used in this study. (DOCX)

S2 Table Segregation of mutant phenotypes in reciprocal crosses between PA64 and *sui1-4* mutant. (DOCX)
