# Supplementary figures and images for: Phosphatidylserine Synthase Controls Cell Elongation Especially in the Uppermost Internode in Rice by Regulation of Exocytosis
Source: PLoS One. 2016 Apr 7;11(4):e0153119. doi: 10.1371/journal.pone.0153119 (PMC4824389; doi:10.1371/journal.pone.0153119)

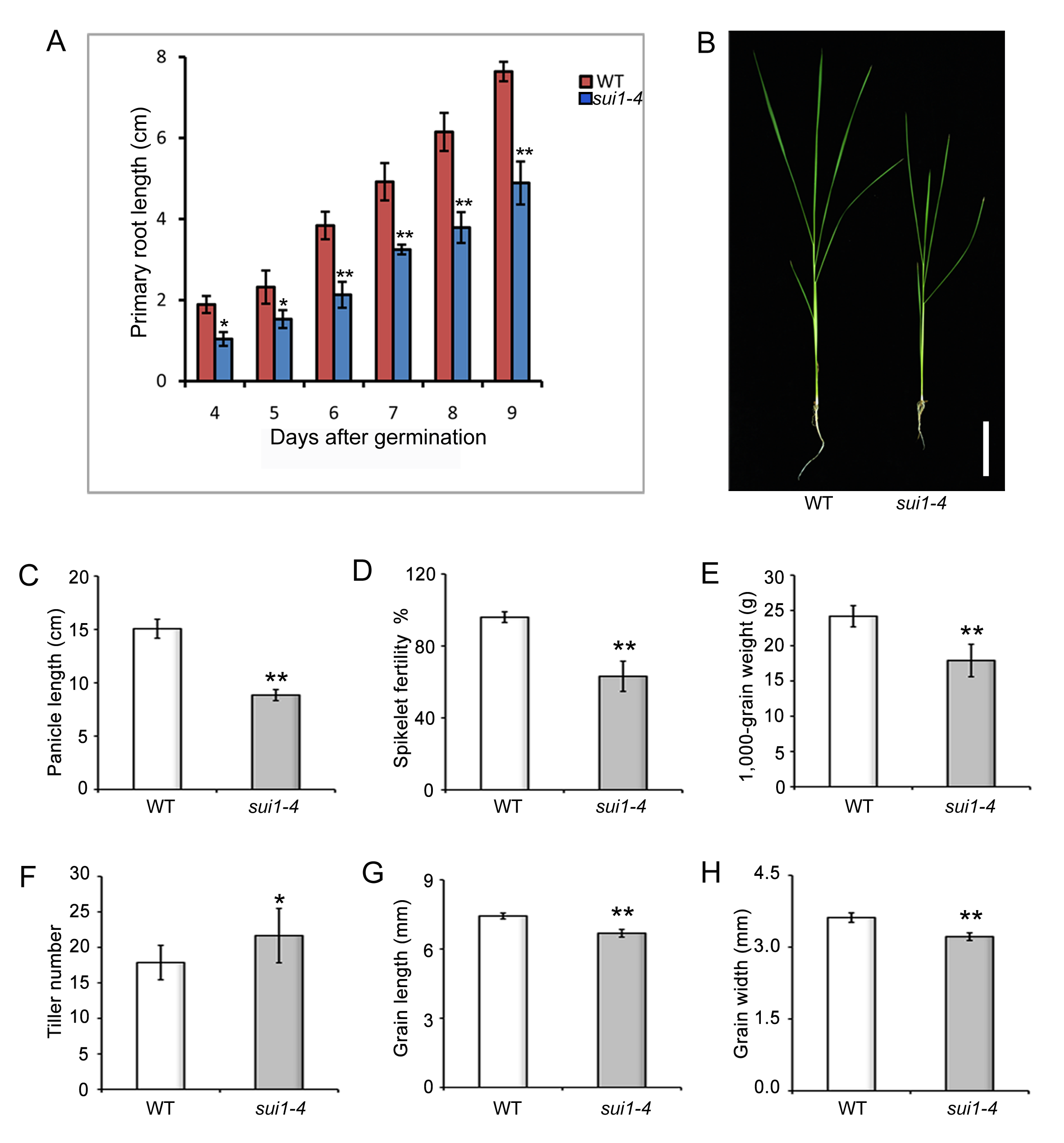

Supplement: S1 Fig — (A) Comparison of primary root growth kinetics in WT and sui1-4 mutant seedlings. Each value represents a mean ± s.d. of 25 seedlings. (B) Comparison of four-weak-old seedings of WT and sui1-4 mutant.(C) to (H) Differences in panicle length (C), spikelet fertility (D), 1000-grain weight (E), tiller number (F), grain length (G), and grain width (H). Data are means ± standard error. Significance was determined by Student’s t-test (*0.01<p<0.05; **p<0.01). (TIF) [file pone.0153119.s001.tif]

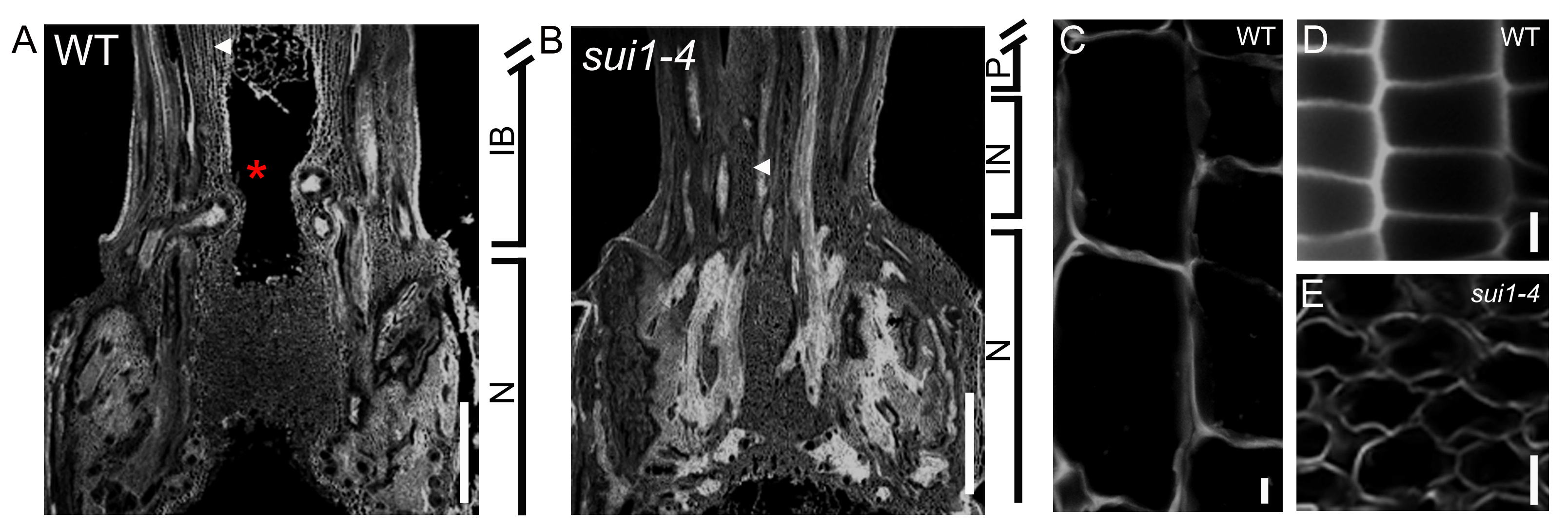

Supplement: S2 Fig — Cell walls were stained with calcofluor white, a non-specific dye for β-glucanblue. The uppermost node and partially attached internode of WT plants (A) and entire uppermost internode of sui1-4 plants (B) are shown. WT cells were well organized at the internode base (D) or elongated in the elongation zone (C), as indicated by white arrowheads in (A), compared to the disorganized, small cells with large intercellular spaces in the sui1-4 mutant (E), as indicated by white arrowheads in (B). Red asterisk indicates an internode cavity. IB, internode base; N, node; IN, internode; P, panicle. Scale bars are 0.5 mm in (A) and (B) and 15 μm in (C) to (E). (TIF) [file pone.0153119.s002.tif]

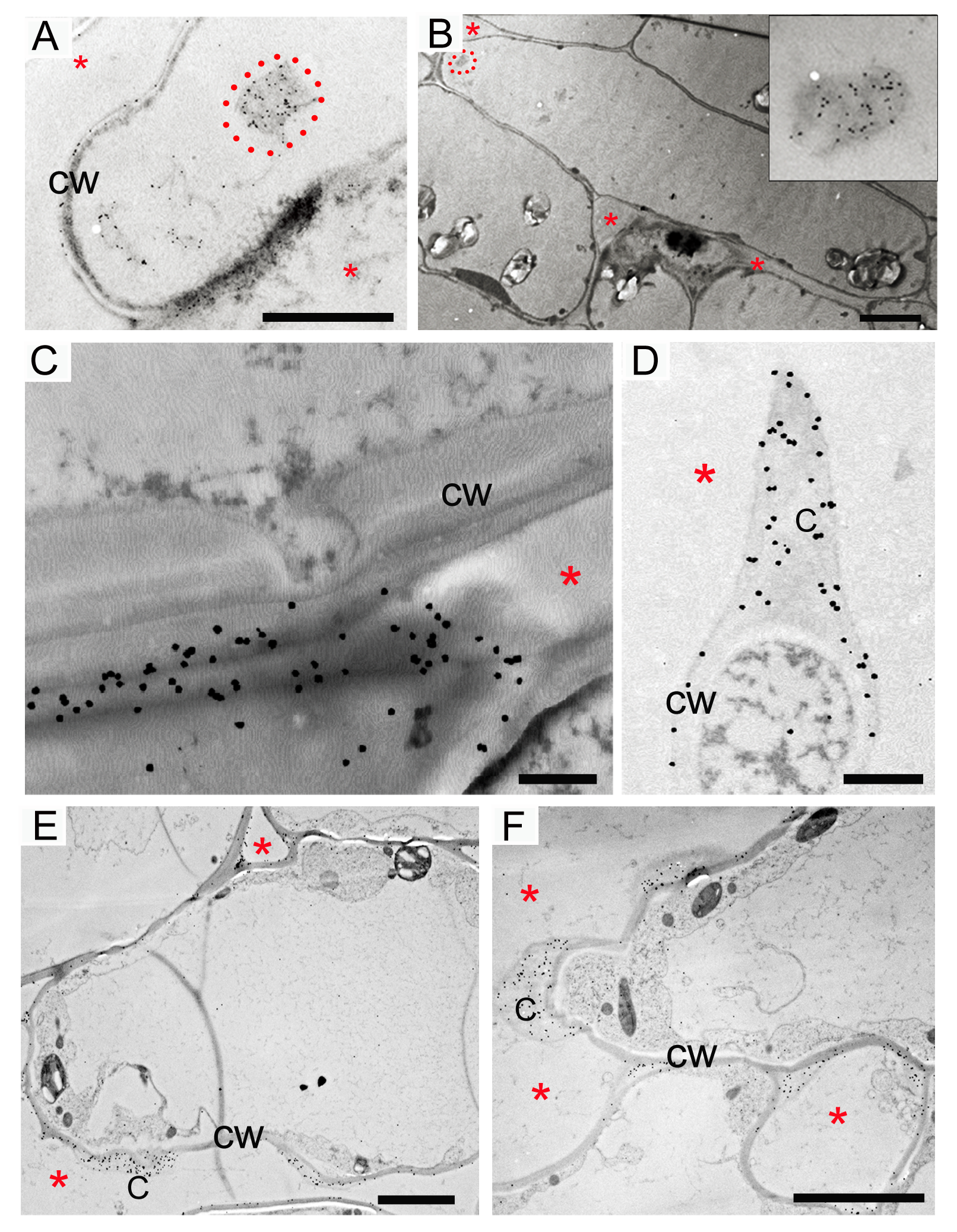

Supplement: S3 Fig — (A) and (B) JIM7-tagged pectin clumps (red dotted circle) inside the cytoplasm. Inset in (B): magnification of selected pectin clumps (red dotted circle). CW, cell wall. Scale bars: 2 μm. In all panels, red asterisks denote intercellular spaces. (C) JIM7-tagged pectin in the cytoplasmic flow or deposited ahead of the flow. Scale bar: 300 nm. (D) JIM7 signal distributed in the clump (C). Scale bar: 1 μm. (E) and (F) JIM7-tagged pectin clumps on immunoelectron microscopy of sections cut from a sample frozen under high pressure. Scale bars: 2 μm. (TIF) [file pone.0153119.s003.tif]

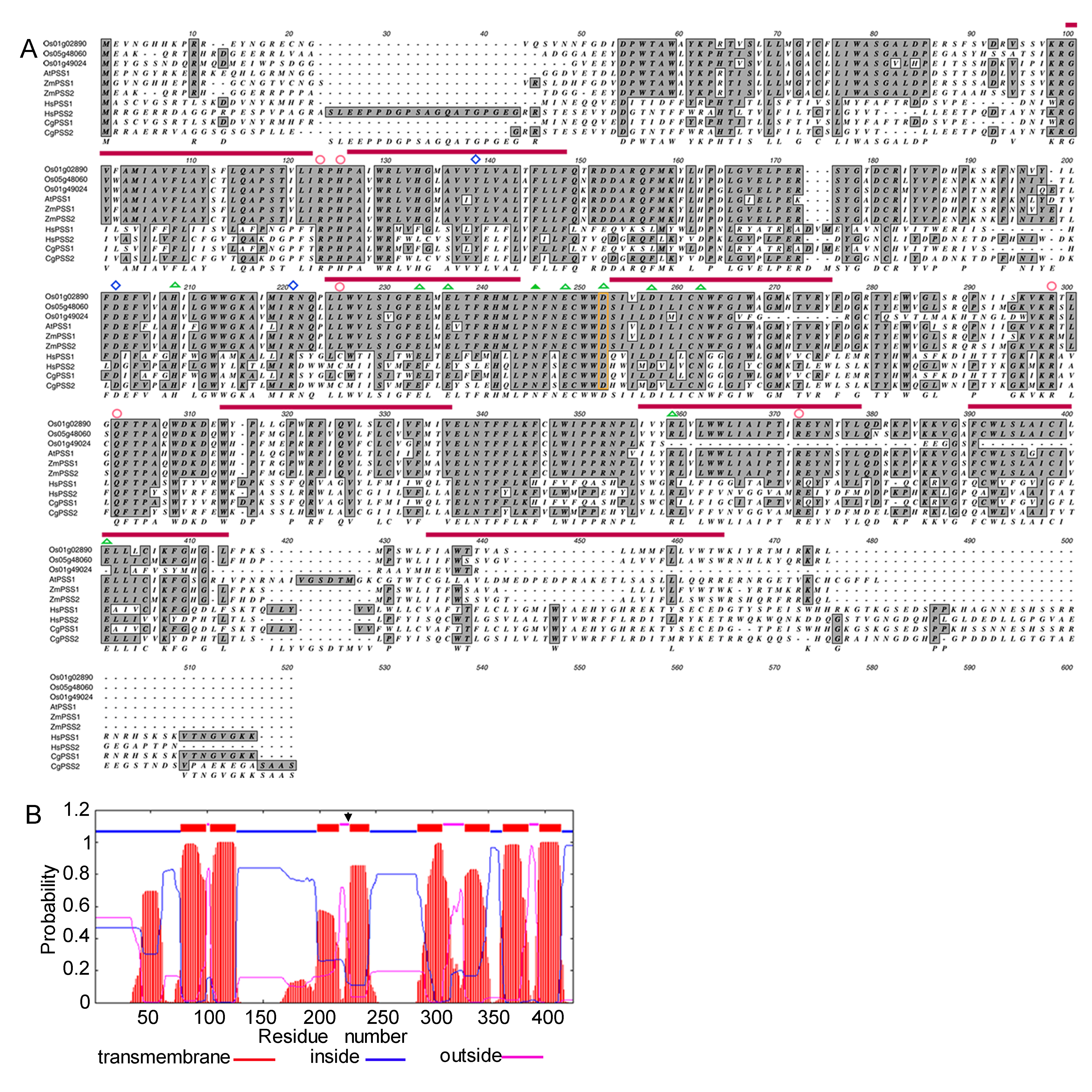

Supplement: S4 Fig — (A) Multiple sequence alignments of the deduced amino-acid sequence of OsPSS-1 and its homologs. The beginning and ending sites of each domain are indicated above the sequences. Eight transmembrane domains were predicted in OsPSS-1 (red lines). Amino-acid residues critical for catalytic activity (open triangles), free serine binding/recognition (closed triangle), enzyme regulation (open circles), and enzyme production and/or stability (diamonds) are highly conserved. The amino acid (boxed by yellow line) mutation at position 225 (Asp→Val) in the sui1-4 mutant is crucial for enzyme action or maintenance of the structure required for serine base-exchange activity. Accession numbers used for this alignment are given in Materials and Methods of the main text. (B) TMHMM v2.0 topology prediction for OsPSS-1. Eight transmembrane domains were predicted, with both the N terminus and a short C terminus facing the cytosol. Black arrow, position of the mutation in the sui1-4 mutant. (TIF) [file pone.0153119.s004.tif]

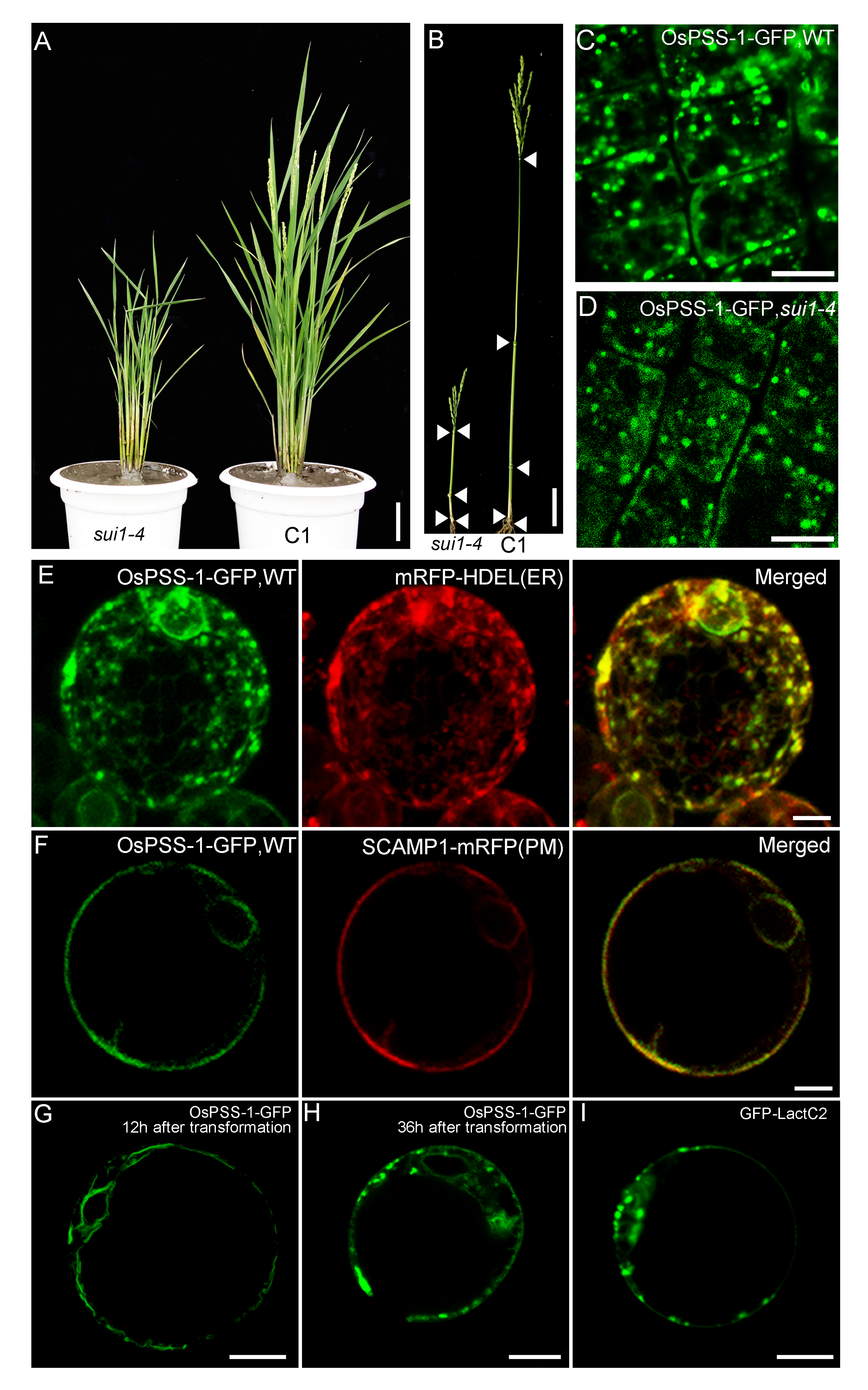

Supplement: S5 Fig — (A) and (B) The 35S promoter-driven OsPSS-1-GFP transgene rescues the phenotype of the sui1-4 mutant. C1 denotes plants from T1 transgenic lines. In (B), white arrowheads indicate each node. Scale bars: 10 cm. (C) and (D) Confocal microscopy indicates that OsPSS-1-GFP is localized to the membrane network and to punctate structures in root epidermal cells of WT (C) and sui1-4 (D) transgenic seedlings. Scale bars: 10 μm. (E) and (F) Confocal microscopy shows that OsPSS-1-GFP (green) colocalizes with the endoplasmic reticulum (ER) (E) and plasma membrane (PM) (F) markers in protoplasts from WT plants. Scale bar: 3 μm. (G) to (I) Subcellular localization of OsPSS-1-GFP (12 h (G) and 36 h (H) after transformation) and GFP-LactC2 12 h after transformation (I) in Arabidopsis protoplasts. (TIF) [file pone.0153119.s005.tif]

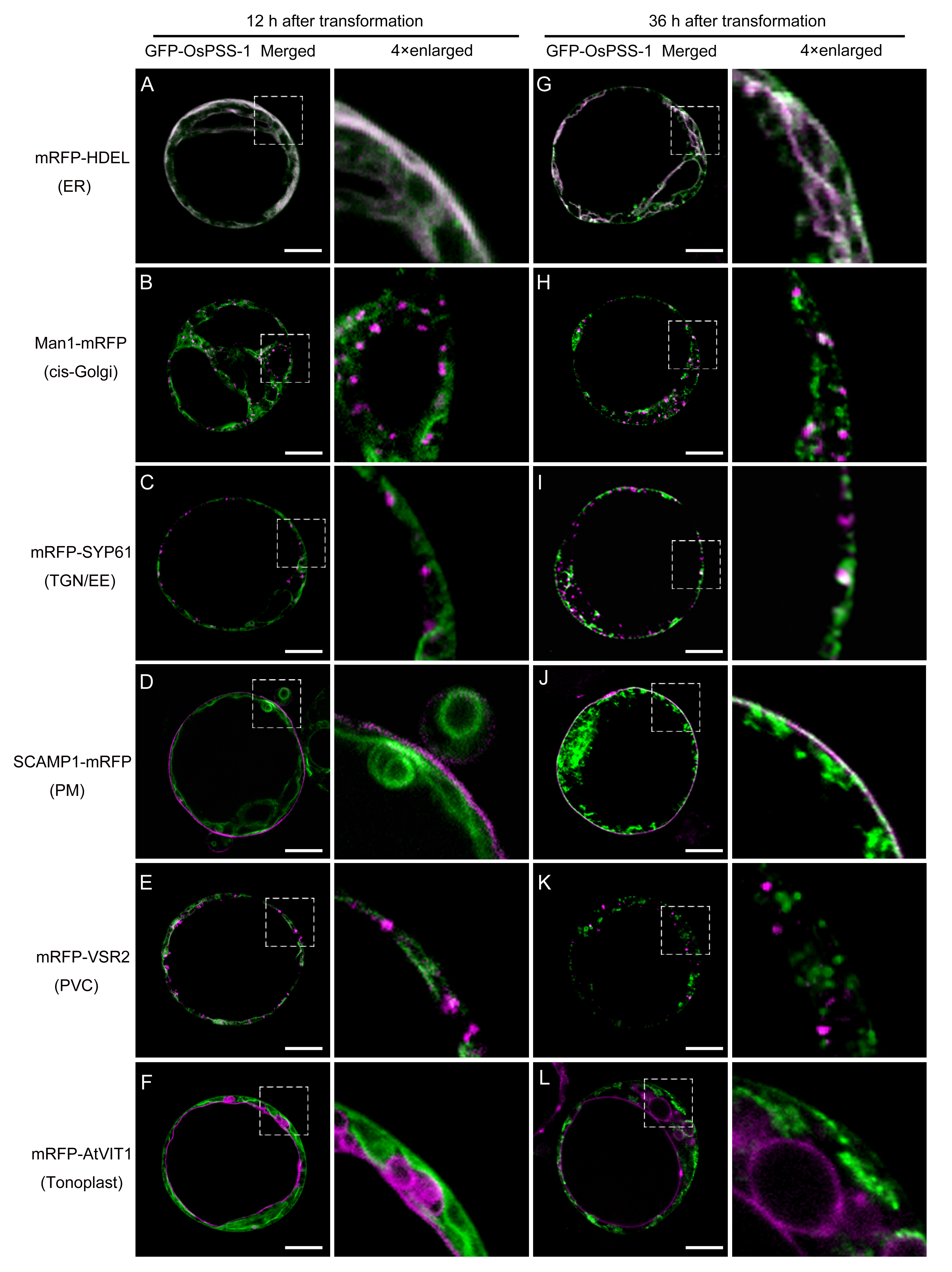

Supplement: S6 Fig — (A) to (F) Confocal microscopy of the distributions of GFP-OsPSS-1 (green) and the indicated markers (magenta) 12 h after transformation. PM, plasma membrane; PVC, prevacuolar compartment; TGN/EE, trans-Golgi network/early endosome. Scale bars: 10 μm. (G) to (L) Confocal microscopy of the distributions of GFP-OsPSS-1 and the indicated markers 36 h after transformation. Scale bars: 10 μm. (TIF) [file pone.0153119.s006.tif]

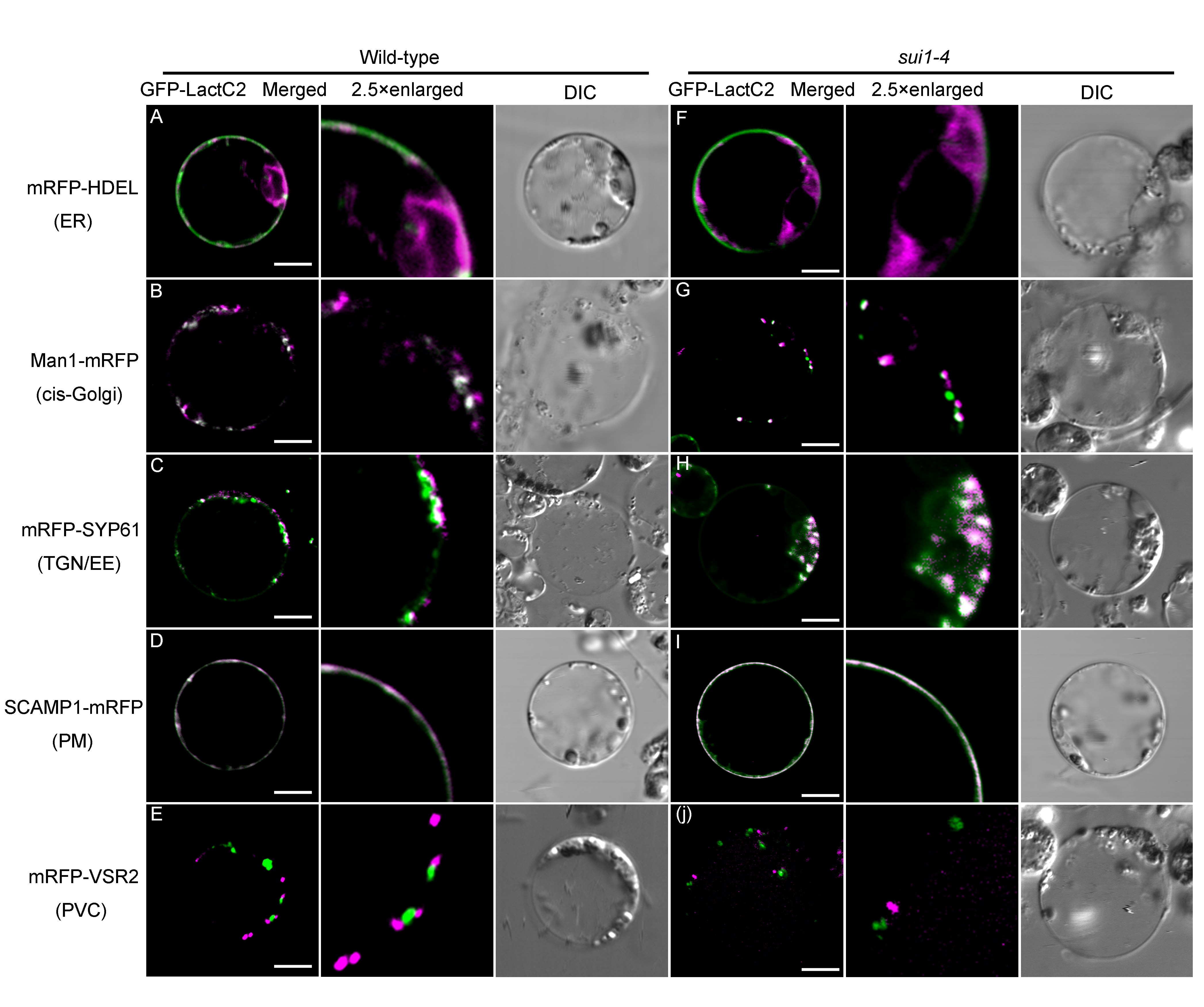

Supplement: S7 Fig — Confocal microscopy reveals the same subcellular localization pattern in wild-type ((A) to (E)) and sui1-4 ((F) to (J)) protoplasts (green, signal from GFP; magenta, signal from RFP). DIC, differential interference contrast; ER, endoplasmic reticulum; PM, plasma membrane; PVC, prevacuolar compartment; TGN/EE, trans-Golgi network/early endosome. Scale bars: 5 μm. (TIF) [file pone.0153119.s007.tif]

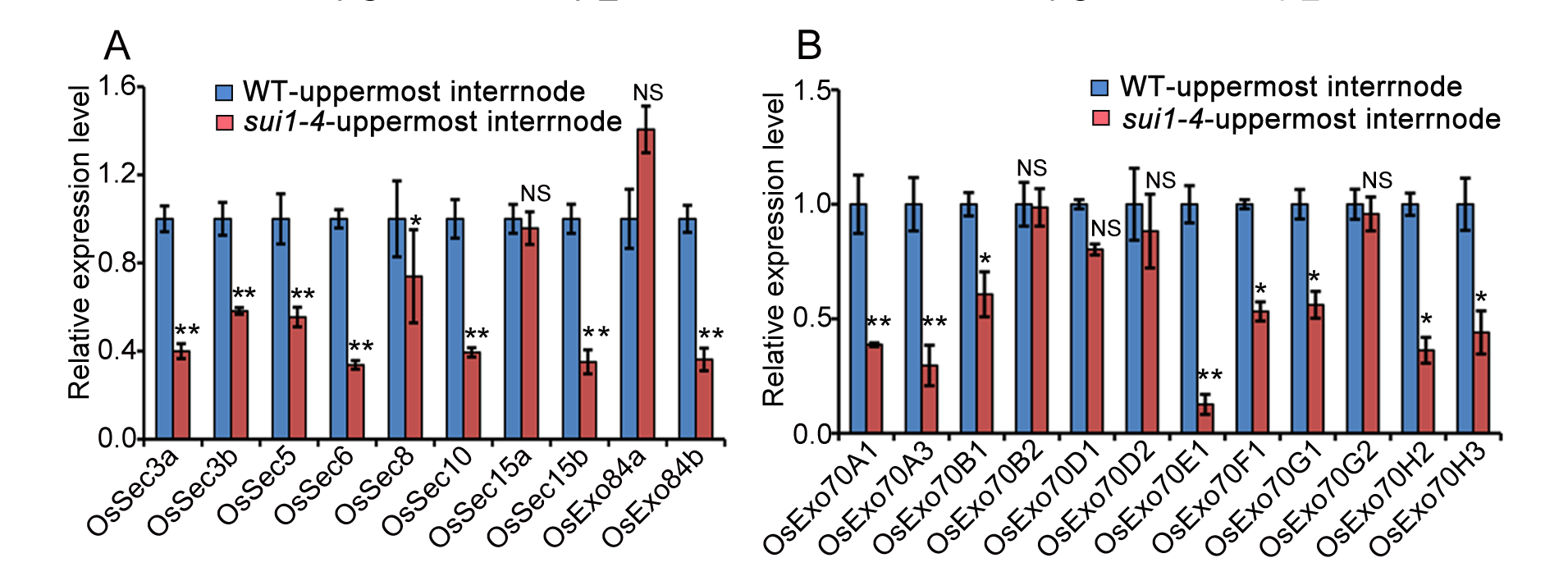

Supplement: S8 Fig — (A) and (B) qRT-PCR of the genes encoding exocyst complex subunits in the uppermost internode of wide type and sui1-4. Significant differences were determined with Student’s t-test (*0.01<P<0.05; **P<0.01; NS, not significant). (TIF) [file pone.0153119.s008.tif]
