# Supplementary material for: Phosphatidylserine Synthase Controls Cell Elongation Especially in the Uppermost Internode in Rice by Regulation of Exocytosis
Source: PLoS One. 2016 Apr 7;11(4):e0153119. doi: 10.1371/journal.pone.0153119 (PMC4824389; doi:10.1371/journal.pone.0153119)
Supplement: S1 Table — (DOCX) [file pone.0153119.s009.docx]

**Supplemental Table 1. Primers used in this study**

| **Use** | **Primer name** | **Sequence** |
| --- | --- | --- |
| **Fine mapping** | IN1-F | AGGGGAAGAAAAACCTGACC |
|  | IN1-R | CCGCGTGCAGATAAAGTACA |
|  | WS6-F | ATGATTGCTGGTCCACAGGT |
|  | WS6-R | TAGCTGCTGGTGAGTTGAGC |
|  | WS7-F | TGCTTTACTTGAGCAACGTAGG |
|  | WS7-R | GAGTTGGCGGAAACATGAAT |
|  | WS10-F | AGCCTGACTCAAGCCATACC |
|  | WS10-R | ACAACTTGATTCATTTTCTTCGT |
|  | Jws11-F | TCCATGTCAGTGAAAATCCAA |
|  | Jws11-R | CGTATACGACAGACAGGGAGGT |
|  | Jws14-F | ATGAATTTAAGTCGGTCGGTGT |
|  | Jws14-R | AACCAAGAGAAAGAAGAAAATGC |
|  | Jws15-F | TGTCCCTGTAGTGGCATTTG |
|  | Jws15-R | TCTGGAGTTCACCCCACAAG |
| **Genomic DNA cloning and RNA interference** | OsPSS-1-HindIII-F | CCGGCGCGCC**AAGCTT**TTGCGA  TGCTCTGTTTTATGCT |
|  | OsPSS-1-PstI-R | GGGGATCCGTCGAC**CTGCAG**CC  TATCATCAATCAGCCAACCA |
|  | OsPSS-1-RNAi1-  KpnI-F | TTACTTCTGCACTA**GGTACC**GGA  GGTCAATGGTCATCACAAA |
|  | OsPSS-1-RNAi1-  KpnI-R | TAGAGCTCAGGCCT**GGTACC**TGG  GCAATAACAAACTCATCAA |
|  | OsPSS-1-RNAi2-  BamHI-F | GAATTCCCGG**GGATCC**GGAGGTC  AATGGTCATCACAAA |
|  | OsPSS-1-RNAi2-  BamHI-R | CGTAGTCGAC**GGATCC**TGGGCA  ATAACAAACTCATCAA |
| **Binary vector construction** | OsPSS-1-Pro-EcoRI-F | CCATGATTAC**GAATTC**TCATCCC  ACCCTTTATGCGAA |
|  | OsPSS-1-Pro-NcoI-R | CTCAGATCTA**CCATGG**GGTGATA  ATTCTTAGCAGCTTAT |
|  | 1305OsPSS-1-GFP-  SmaI-F | TCGAGGACCGGT**CCCGGG**ATGG  AGGTCAATGGTCATCACA |
|  | 1305OsPSS-1-GFP-  SmaI-R | CACCATGGATCC**CCCGGG**TAGC  CTTTTCCTTATCATTG |
| **Transient expression vector construction** | OsPSS-1-GFP-  BamHI-F | CGGTCCCGGG**GGATCC**ATGGAG  GTCAATGGTCATCACA |
|  | OsPSS-1-GFP-  BamHI-R | TGCTCACCAT**GGATCC**TAGCCTT  TTCCTTATCATTG |
|  | GFP -OsPSS-1  BgIII-F | CGAGCTGTAC**AGATCT**ATGGAGG  TCAATGGTCATCACA |
|  | GFP -OsPSS-1  BgIII-R | GGCCGCTTTA**AGATCT**TAGCCTT  TTCCTTATCATTG |
|  | C2domain-BgIII-F | CGAGCTGTAC**AGATCT**TGCACTG  AACCCCTAGGCCTGAA |
|  | C2domain-BgIII-R | GGCCGCTTTA**AGATCT**CTAACAG  CCCAGCAGCTCCACT |
| **RT-PCR and qRT-PCR** | RTcF1 | CGTGATGATGCTAGGCAATTTA |
|  | RTcR1 | CATCAAAAAGGATCTCATAAACA |
|  | ActinF | TGGAACTGGTATGGTCAAGGC |
|  | ActinR | AGTCTCATGGATACCCGCAG |
|  | qOsPSS-1-F | GGTATTTGGGCTGGAATGAAGA |
|  | qOsPSS-1-R | ACCACTCATCTTTGTCCCACTG |
|  | qOsExo70A1-F | GTTGCTGAAATCCTGTTGCC |
|  | qOsExo70A1-R | TACGCTCTTGTTTTCCTTCA |
|  | qOsExo70A3-F | GCAAGCCCTACAAAATAACT |
|  | qOsExo70A3-R | TGAAACCCCACTGCTGCTAA |
|  | qOsExo70B1-F | ACACCCTTGGTTCCTCTATC |
|  | qOsExo70B1-R | CACGGAGATAGTTCATCACAT |
|  | qOsExo70B2-F | TGCGACTTGAGGATGTGAGA |
|  | qOsExo70B2-R | AACTGTTTAGCCTGGGTGGG |
|  | qOsExo70D1-F | CTCCTGAGGGCAATGAGAAT |
|  | qOsExo70D1-R | GTGCCGCATCCTTGTAGAGT |
|  | qOsExo70D2-F | GACGCCACTTGCTGCTCATA |
|  | qOsExo70D2-R | CGCTTCAAGTAATCATCACCAA |
|  | qOsExo70E1-F | CCACCGGCGAACTTTTGACA |
|  | qOsExo70E1-R | GTGGCGACTGAAAATGATGC |
|  | qOsExo70F1-F | ATCGTCCGAGAAGCTGTTCC |
|  | qOsExo70F1-R | TCCCCACCGGGCAACGCTCT |
|  | qOsExo70G1-F | TCTTGAGTCCGAGCGACAGC |
|  | qOsExo70G1-R | AAACGCAGGAGCTTAATGGG |
|  | qOsExo70G2-F | TCAAGAGGGTGGTGGATGGT |
|  | qOsExo70G2-R | GTGAGCACAGAGCGATACGG |
|  | qOsExo70H2-F | TGAGGCTGGTCGGTTTCTGT |
|  | qOsExo70H2-R | TGCCCTACAACACCAGCGTC |
|  | qOsExo70H3-F | AGCCCTTGACCGCATAAACC |
|  | qOsExo70H3-R | TGTTGTTCGCCATGAAGAGG |
|  | qSec3a-F | TGTCCCAAGCAAAGGATGAA |
|  | qSec3a-R | AAATGCGTAACCACTCATCC |
|  | qSec3b-F | TGTGGAGATGGCTATGTGGG |
|  | qSec3b-R | GTCTTTCTGAATGGCAACTT |
|  | qSec5-F | CGTGGGAACATTAGAAAAGG |
|  | qSec5-R | CCTCCATTGACTTGTAAAGC |
|  | qSec6-F | GTTGCTACGAACTTTCATCA |
|  | qSec6-R | AACGCTGACAGTTTGAAGGA |
|  | qSec8-F | CATACCTGACCAAGGGATTT |
|  | qSec8-R | ACAAAAGCCAATAGCCCATC |
|  | qSec10-F | CGATGGCAGAATGTGCTAAA |
|  | qSec10-R | CCTCCTCACCAAGAACAACT |
|  | qSec15a-F | GATTCCCGTGTCACAAGCAG |
|  | qsec15a-R | AAATCCTCGTAATGAAGTCT |
|  | qSec15b-F | CTCATTCCACTCGCCTCCAT |
|  | qSec15b-R | CTTCATTTTCACGATGCTCC |
|  | qExo84a-F | AGACCGACAACTTCGACCCC |
|  | qExo84a-R | CATAAACACTTCTACGCATC |
|  | qExo84b-F | CCAACTGGCATTCGTCCTTT |
|  | qExo84b-R | TCATCCATTGTAGAACCACC |
