# Supplementary material for: Phosphatidylserine Synthase Controls Cell Elongation Especially in the Uppermost Internode in Rice by Regulation of Exocytosis
Source: PLoS One. 2016 Apr 7;11(4):e0153119. doi: 10.1371/journal.pone.0153119 (PMC4824389; doi:10.1371/journal.pone.0153119)
Supplement: S2 Table — (DOCX) [file pone.0153119.s010.docx]

**Supplemental Table 2. Segregation of mutant phenotypes in reciprocal**

**crosses between PA64 and *sui1-4* mutant.**

| Cross combination Normal Plants Mutant Plants  *X*^2^_3:1_^a^ |
| --- |
| *sui1-4/*PA64 F2 389 112 1.86  PA64*/sui1-4* F2 412 141 0.07 |
| _a_ Value for significance at *P* = 0.05 and 1df is 3.84. |
